# Advanced structural characterization of condensed tannin evolution markers in long-aged red wines under different closure oxygen permeabilities

**DOI:** 10.1016/j.crfs.2026.101528

**Published:** 2026-08-14

**Authors:** Lantomalala Elsa Razafindrabenja, Emmanuelle Meudec, Arnaud Verbaere, Soline Caillé, Nicolas Galy, Dimitri Tixador, Christophe Loisel, Nicolas Sommerer, Laetitia Mouls

**Affiliations:** a SPO, Univ Montpellier, INRAE, Institut Agro, Montpellier, France; b INRAE, PROBE Research Infrastructure, PFP Polyphenol Analysis Facility, Montpellier, France; c Diam Bouchage, Céret, France

**Keywords:** Condensed tannins, Polyphenols, Depolymerization, UHPLC–UHRMS, Molecular networking, Oxygen transfer rate, Evolution marker

## Abstract

Understanding the long-term evolution of condensed tannins (CTs) in wine remains challenging due to the molecular complexity of aged wines. In this study, an advanced analytical workflow combining tannin-fraction isolation, thioglycolysis, UHPLC–UHRMS with AcquireX™ data-dependent acquisition, and Feature-Based Molecular Networking (FBMN) driven by the t-distributed stochastic neighbor embedding (t-SNE) algorithm was applied for the first time to wines. The studied wines are authentic red wines aged for 17 years. This exceptionally rare sample set, stored under four closures of differing oxygen permeability, provided a unique opportunity to assess the applicability of this workflow for the in-depth structural characterization of condensed tannin evolution markers in a complex wine matrix. Using this approach, 139 evolution markers were annotated. Among these, 122 markers were assigned to levels 2–3, while 17 markers were assigned to level 4. Overall, 72 of the 139 annotated markers were previously unreported, including 55 markers within the levels 2–3 group and 17 within the levels 3–4 group. These markers arose from interactions between tannins and anthocyanins, pyranoanthocyanins, and aromatic aldehydes with high oxidation levels of up to 5. Exploratory univariate (ANOVA) and multivariate statistical methods including Principal Component Analysis (PCA) and Hierarchical Cluster Analysis (HCA) revealed correlative trends between marker abundance and closure oxygen permeability within this specific sample set. Overall, this work demonstrates the power of this transferable analytical workflow for comprehensive structural characterization of condensed tannin evolution markers in complex matrix form long-aged red wines and provides new molecular insights into their chemical evolution.

## Highlights

• First application of thioglycolysis, AcquireX™ + FBMN/t-SNE workflow to tannin fractions isolated from authentic 17-year-old red wines.
• Comprehensive structural annotation of 139 condensed tannin evolution markers, including 72 newly proposed.
• Molecular networking revealed chemically coherent families of evolution markers derived from different tannin-related interactions.
• Within this sample set, correlative trends were observed between closure oxygen permeability and advanced oxidation markers relative abundance.

## Graphical abstract

**Figure:**
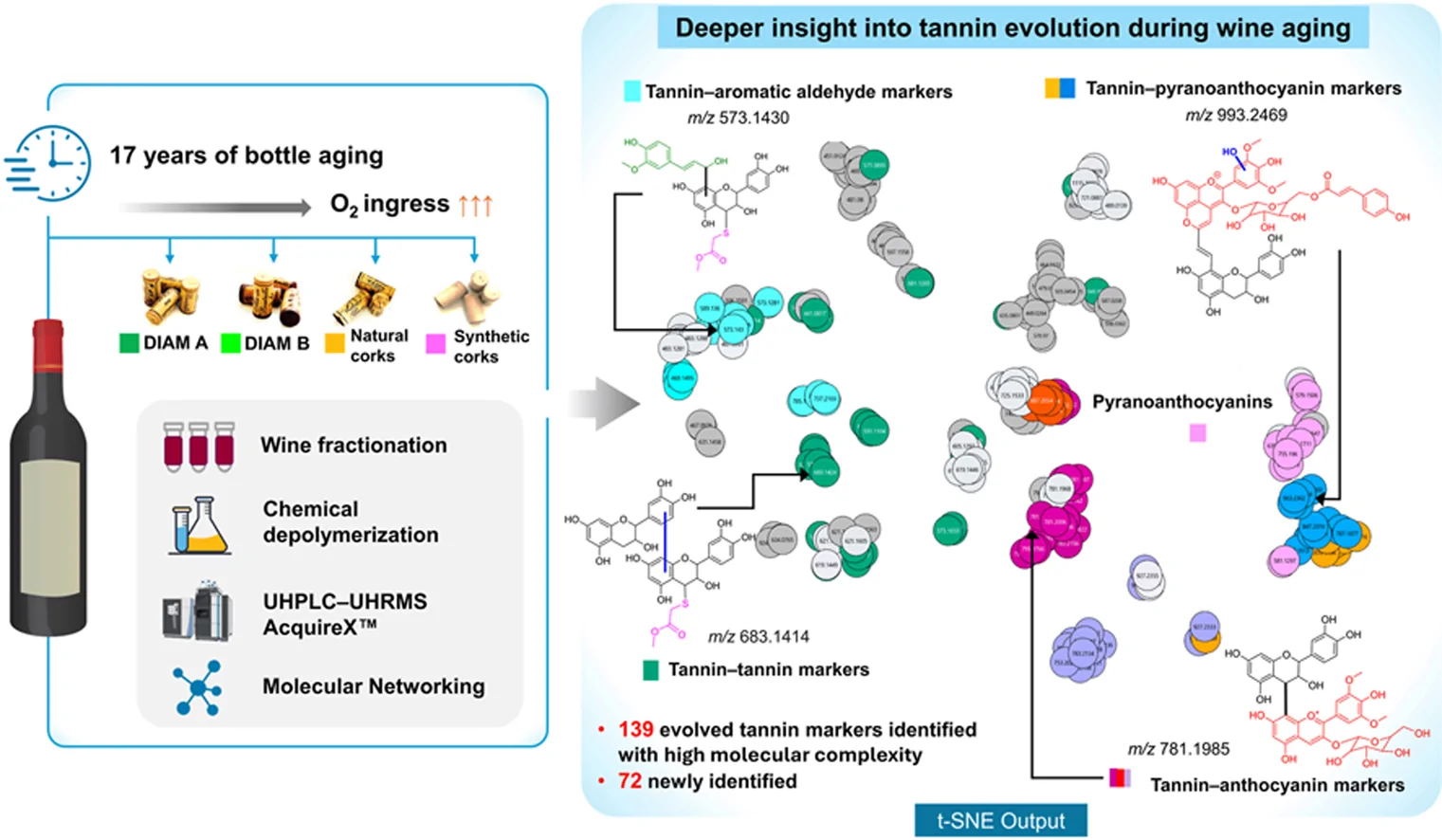

## 1 Introduction

Red wines designed for bottle aging are complex matrices, and their quality is highly governed by their chemical composition and its subsequent evolution. These hydroalcoholic systems contain a wide variety of compounds that collectively determine both chemical stability and sensory characteristics. Among these constituents, phenolic compounds are key contributors to its aging capacity (Zhang et al., 2020).

Within this phenolic fraction, condensed tannins (CTs), also called procyanidins, are formed by the polymerization of flavanol units, including catechins and epicatechins, linked by C_4_–C_8_ (or C_4_–C_6_) bonds (Type B), or with additional interflavan ether-linkage C_2_–*O*–C_7_ (or C_2_–*O*–C_5_) (Type A) (Dixon et al., 2005; Ferreira and Bekker, 1996). During wine aging, beyond their high reactivity, CTs undergo extensive structural modifications through oxidative and non-oxidative reactions. In fact, CTs participate in a wide range of interactions, contributing to CT chain polymerization (Poupard et al., 2011), as well as reactions with bisulfite, a well-known nucleophile (Ma et al., 2018), with anthocyanins *via* condensation reactions (Fulcrand et al., 1996; Remy et al., 2000), and with pyranoanthocyanins (Cruz et al., 2008; He et al., 2006; Marquez et al., 2013). They can further associate with aroma compounds (Nikolantonaki et al., 2010, 2012; Suc et al., 2021) and interact with macromolecules such as polysaccharides and proteins (Hagerman and Butler, 1981; Harbertson et al., 2014). All these processes collectively modulate the organoleptic properties of wine, particularly its astringency, bitterness, and color stability (Noble, 1994; Peleg et al., 1999; Salas et al., 2004).

Several studies have characterized oxidation markers, using simplified matrices such as model solutions or purified grape seed extracts, providing foundational insights into their chemical diversity (Tanaka et al., 2001; Poupard et al., 2008; Mouls and Fulcrand, 2012, 2015; Suc et al., 2021; Wong-Paz et al., 2021; Deshaies et al., 2022). However, transferring these findings to real wine matrices remains challenging, and the molecular characterization of CT evolution in complex wines remains incomplete. This limitation mainly arises from the extreme diversity and complexity of reaction products, the dynamic reactivity of flavan-3-ol units, together with the occurrence of numerous low-abundance transformation products. In addition, co-elution of structurally related compounds and the limited resolving power and sensitivity of conventional analytical strategies further complicate their comprehensive characterization (Mouls et al., 2011; Yu et al., 2020).

In this analytical context, high-resolution mass spectrometry has emerged as a powerful analytical strategy for improving the detection and structural characterization of CT-derived transformation products. Recently, advanced analytical workflows combining acid-catalyzed depolymerization (Matsuo et al., 1984), with ultra-high-performance liquid chromatography coupled to ultrahigh-resolution mass spectrometry (UHPLC–UHRMS), using AcquireX*™* deep scan workflow (Geng et al., 2021), together with MS/MS-based Molecular Networking (MN) (Xie et al., 2020) have been developed to overcome these limitations (Razafindrabenja et al., 2025). Applied to grape seed CT oxidation markers, this approach significantly improves MS/MS coverage and annotation efficiency by organizing spectral features according to their fragmentation similarity, enabling faster structural characterization of oxidation markers (Razafindrabenja et al., 2025).

While this workflow has previously been validated on simplified grape-seed condensed tannin extracts, its applicability to the structural characterization of CT evolution markers in wine matrices remains to be demonstrated. The present study reports the first application of this combined analytical workflow to red wines (70% ‘Cabernet-Sauvignon’ and 30% ‘Merlot’) aged for 17 years, enabling expanded detection and in-depth structural characterization of CT evolution markers. Our hypothesis is that the long-term wine matrix undergoes progressive transformations, giving rise to evolution markers that have not yet been finely characterized. This ultimately provides deeper insights into the molecular reactions governing CT transformation in complex wines. Such aged wines are exceptionally rare, which have undergone progressive condensed tannin transformations over time, and offer a unique opportunity to investigate tannin evolution inaccessible through conventional short-term studies.

A recent metabolomic study conducted on the same exceptionally aged wines investigated the influence of bottle closures on sensory properties and tannin evolution using an UHPLC–UHRMS metabolomic approach (Razafindrabenja et al., 2026). This metabolomic approach primarily provided insights into global chemical variations but did not enable the detailed structural characterization of CT evolution markers. The present study therefore aimed to structurally characterize these markers through the analysis of their MS/MS fragmentation patterns, providing an additional level of confidence and structural insight into the molecular features. A complementary exploratory objective was to examine whether the structurally annotated markers displayed abundance trends associated with differences in closure oxygen permeability within this specific sample set, thereby providing a complementary chemical interpretation of the metabolomic trends previously reported for the same samples.

For this purpose, our approach did not examine whole wines directly. Instead, we focused specifically on CTs by isolating their enriched fraction, a crucial step to reduce matrix complexity and ensure that observed differences arise directly from tannin chemistry. The collected sub-fraction was then subjected to thioglycolysis, followed by data acquisition. MN were generated from the processed MS/MS features using MZmine (Schmid et al., 2023) and MetGem softwares (Olivon et al., 2018), allowing the visualization and comparison of evolution markers groups across samples.

## 2 Materials and methods

### 2.1 Chemical reagents and materials

Standards of (+)-catechin (≥98%), (−)-epicatechin (≥90%), procyanidin B1 and B2 (≥90%), and malvidin-3-*O*-glucoside chloride (≥95%) were purchased from Sigma-Aldrich (Steinheim, Germany) and Extrasynthese (Genay, France). Thioglycolic acid (≥98%) and hydrochloric acid were purchased from Sigma-Aldrich (Saint-Quentin Fallavier, France). Ammonium acetate (≥97%) was acquired from Sigma-Aldrich (Saint Louis, USA). LC-MS-grade methanol (MeOH) (≥99%) was acquired from VWR Prolabo (Fontenay-sous-Bois, France). LC-MS-grade formic acid (≥99%) and LC-MS-grade Acetonitrile (≥99%) were purchased from Biosolve (Dieuse, France). Ultrapure water was produced using a Milli-Q water system (Millipore, Bedford, MA, USA). Fractogel Toyopearl® HW-50F was from Tosoh Bioscience, ethanol (≥99%) from Merck/Sigma-Aldrich (Saint-Quentin Fallavier, France), and acetone (≥99%) from VWR chemicals (Pennsylvanie, USA).

### 2.2 Wine samples

Red wines (70% ‘Cabernet-Sauvignon’ and 30% ‘Merlot’), obtained from Bordeaux wineries (Graves AOC), were selected for their high-aging potential, based on grape composition, ripeness, winemaking process and sensory quality as detailed previously (Pons et al., 2022). Wines were produced following standard winemaking protocol, matured in French oak barrels (new to 2 years old). Free SO_2_ was adjusted to the wines in vat two days before bottling and no copper fining was applied. Bottling occurred between April and July 2008 in standard 750 mL green glass bottles (Saint-Gobain Emballage, Cognac, France), which were inverted 1 h after filling. The overall bottling procedure has been previously described (Pons et al., 2022). Bottles were stored for 17 years under controlled temperatures at 17 ± 1.5 °C and relative humidity maintained within the recommended range of 40–65% (Razafindrabenja et al., 2026).

### 2.3 Wine closures

Four closure types were selected according to the oxygen transfer rate (OTR) at the beginning proposed by the different manufacturers as described previously (Pons et al., 2022): synthetic cork made of recyclable thermoplastic elastomers, natural cork and microagglomerated corks (DIAM A and B), as presented in Table S1 in Supplementary Materials. Synthetic corks showed an initial OTR of 4.6 mg/year, while DIAM A and DIAM B microagglomerated corks had initial OTRs of 0.3 and 0.4 mg/year, respectively. Natural corks showed highly variable OTRs due to intrinsic structural heterogeneity (Crouvisier-Urion et al., 2018). After 17 years of storage, OTR of some closures were determined by coulometry, under the measurement conditions previously reported (Suhas et al., 2025). Therefore, values for DIAM A and DIAM B were 0.22 and 0.66 mg/year, respectively. For each closure type, three independent bottles were analyzed and considered as biological replicates. Each bottle represented an independent wine sample and was independently processed for subsequent analysis. A total of 12 wine samples (4 closure types × 3 replicates) were studied.

### 2.4 Tannin fraction isolation by SPE

**Sample and column preparations**: Fractionation procedure, performed independently on three individual bottle samples (biological replicates), was adapted from method described in the previous study (Razafindrabenja et al., 2026). Fractogel HW-50 F resin was suspended in water/0.05% trifluoroacetic acid (TFA) and approximately 6 mL of resulting suspension were packed into 15 mL polypropylene SPE cartridges with 20 μm porosity frits. Cartridges were conditioned with water/0.05% TFA. Subsequently, 5 mL of each wine samples were then applied onto the fractogel columns.

**Separation conditions**: Solvents used for elution were: ethanol (A), water/0.05% TFA (B) and acetone (C). Elution was carried out under a constant pressure of 3.0 psi by sequentially applying 3×5 mL of each solvent in the following order: 15% A/85% B (*v/v*); 30% A/70% B (*v/v*); 35% A/65% B (*v/v*); 40% A/60% B (*v/v*); 50% A/50% B (*v/v*); 60% A/40% B (*v/v*); 30% A/25% B/45% C (*v/v/v*).

**Fraction collection**: collected fractions from 12 wine samples were analyzed by UHPLC–UHRMS, then pooled according to their MS profiles before evaporation and lyophilization. A fraction containing tannins (corresponding to 40%–60% A and 30% A/25% B/45% C) was selected for further processing and stored at −80 °C.

### 2.5 Chemical depolymerization (thioglycolysis)

In this work we used the same thioglycolysis procedure described in the previous works (Razafindrabenja et al., 2025; Razafindrabenja et al., 2026).

### 2.6 UHPLC–UHRMS analysis

All analyses were performed on a Vanquish UHPLC–UHRMS system (Thermo Fischer Scientific, San José, CA, USA) with a UV–visible diode array detector covering the full range of acquisition (190–650 nm) and an Acquity UPLC® HSS T3 C18 analytical column (100 × 1.0 mm i. d.; 1.8 μm particle size) (Waters, Milford, MA, USA). Elution used a gradient of (A) water/formic acid (99/1, *v/v*) and (B) acetonitrile/water/formic acid (79.5/19.5/1, *v/v/v*) at 0.22 mL/min. The elution program was as follows: 0–1 min: 2% B; 1–2.5 min: 2%–12% B; 2.5–5 min: 12% B; 5–10 min: 12%–24% B; 10–13 min: 24%–48% B; 13–18 min: 48%–60% B; 18–23 min: 60%–100% B; 23–25 min: 100% B; 25–26 min: 100%–2% B and 26–30 min: 2% B. Column and injector temperatures were maintained at 35 and 10 °C, respectively. The injection volume was 0.5 μL (Razafindrabenja et al., 2025).

ESI–MS analyses were performed with an Orbitrap Exploris 480 from Thermo Fisher Scientific (San José, CA, USA) with an ESI source using an internal post-source fluoranthene mass calibrant (radical ion at *m/z* 202.0777 in positive ion mode). The instrument was operated in positive ion mode. Source parameters were as follows: ion transfer tube temperature: 280 °C; vaporizer temperature: 300 °C; sheath gas, auxiliary gas and sweep gas: 40, 10 and 2 (arbitrary units), respectively; S-lens RF level: 50%; spray voltage: 3.5 kV (ESI+). The mass range was *m/z* 200–2000. MS full scan resolution was set to 480,000.

ESI–MS/MS analyses were carried out using an AcquireX*™* Deep Scan workflow (Geng et al., 2021). A solvent blank was first injected in full scan mode to generate an automated exclusion list of background ions. A subsequent full-scan UHRMS injection of the sample generated an inclusion list of sample-derived ions. Using these lists, multiple iterative data-dependent acquisition (DDA) injections of sample were performed: only ions in the inclusion list were targeted for MS/MS fragmentation based on signal intensities, while ions in the exclusion list and isotopic peaks of fragmented precursors were excluded. After each MS/MS run, both lists were automatically updated and previously fragmented precursors were added to the exclusion list for the next injection. Standard DDA injection was carried out using the same method. For the DDA method, MS full scans were acquired at 60,000 resolutions; and all MS/MS at 30,000. Dynamic exclusion was applied in custom mode, allowing each precursor ion to be selected once before being excluded for 2s. The isolation window was set to *m/z* 2. Fragmentation was performed by stepped HCD with normalized collision energy of 15%, 30% and 45%.

AcquireX*™* experimental parameters were configured as follows: exclusion override factor of 3; no peak window extension for exclusion or inclusion lists; 50% fragmentation threshold for inclusion; exclusion duration of 1s; preferred ions [M+H]^1+^ and [M+2H]^2+^; automatic isotope addition enabled; minimum intensity thresholds of 2.0 × 10^4^ for both exclusion and inclusion lists. Instrument control, data acquisition and analysis were performed with Xcalibur™ 4.7. (Thermo Scientific, Waltham, MA, USA).

For the structural characterization, a quality control (QC) sample was prepared by combining 75 μL of each depolymerized tannin fraction. The pooled QC sample was analyzed using 10 consecutive iterative MS/MS injections with the AcquireX™ workflow in order to maximize MS/MS coverage.

Before each analytical sequence, a mixture of commercially available phenolic standards (gallic acid, caffeic acid, epicatechin, rutin, quercetin) was analyzed to assess instrument quality control, including interday retention time stability, peak area reproducibility, signal-to-noise ratio, and mass accuracy. These standards cover the main polyphenol classes in wine. Interday quality control results are provided in Supplementary Materials (S2).

### 2.7 Chemometrics

The UHPLC–MS/MS raw data were converted into mzXML files using MSConvert 3, part of the ProteoWizard package (Kessner et al., 2008). Raw files were directly processed using MZmine 4.7.29 software (Schmid et al., 2023). All parameters used for data processing are described in the Supplementary Materials (S3). The aligned feature tables (.csv) and MS/MS spectra files (.mgf), were exported and uploaded to MetGem for the creation of MNs.

The MN were created, visualized and annotated using MetGem 1.5.2 software **(**https://metgem.github.io**)** (Olivon et al., 2018). Parameters used for processing are described in the Supplementary Materials (S4). Features were annotated based on predicted molecular formulas, MS/MS fragmentation patterns and literature comparison.

### 2.8 Statistical analysis

Statistical data analyses were performed using XLSTAT software (2025.2.0.1432 version, Lumivero, Paris). For the statistical evaluation of closure-related trends, features detected by full-scan UHPLC–UHRMS acquisition per biological replicate (n = 3) were used. One-way ANOVA was used to identify markers significantly affected by bottle closure type (α = 0.05, confidence interval = 95%, tolerance = 0.0001). Differences were considered statistically significant at *p*-values ≤ 0.05. As this analysis was exploratory, no multiple-comparison correction was applied. Variables showing differences at *p* ≤ 0.05 were retained as features of interest for further exploration, resulting in 212 variables. Pairwise mean comparisons were performed using Tukey's test. Principal component analysis (PCA) and Hierarchical Cluster Analysis (HCA) were then performed on significant variables. PCA was performed on standardized data using a correlation matrix. HCA was computed using Euclidean distance as the dissimilarity measure and Ward's linkage method. For visualization purposes only a filter (*p* ≤ 0.03) was applied to PCA and HCA plots, resulting in 144 variables (Fig. 7). All interpretive conclusions, however, rest on the full set of 212 variables (provided in Supplementary Table S2).

## 3 Results and discussion

### 3.1 Characterisation of evolution markers in 17-year-old red wines

#### 3.1.1 Tannin fractions

Because wine is an extremely complex matrix containing many interacting compounds, isolating the tannin fraction is required to enable an extensive characterization of the polymeric CTs. The isolation of tannin was carried out, and the concentrations for each sample, reported in Supplementary Materials (S5), ranged from 2.25 to 2.72 g.L^−1^, with minor variations across closure types. Such variations may arise from precipitation of some tannins through interactions with other wine components. Overall, these values fall within the typical range reported for aged red wines (1.0–4.0 g.L^−1^) (Harbertson et al., 2008).

#### 3.1.2 UHPLC*–*MS/MS and molecular networking

Detection and identification of tannin-derived compounds required high-quality MS and MS/MS spectra with well-defined fragment ions. The UHPLC–UHRMS Orbitrap platform enabled high-resolution, accurate-mass analysis with reliable molecular formula assignment. Its iterative fragmentation approach enhances MS/MS coverage of all targeted compounds.

**Fig. 1:**
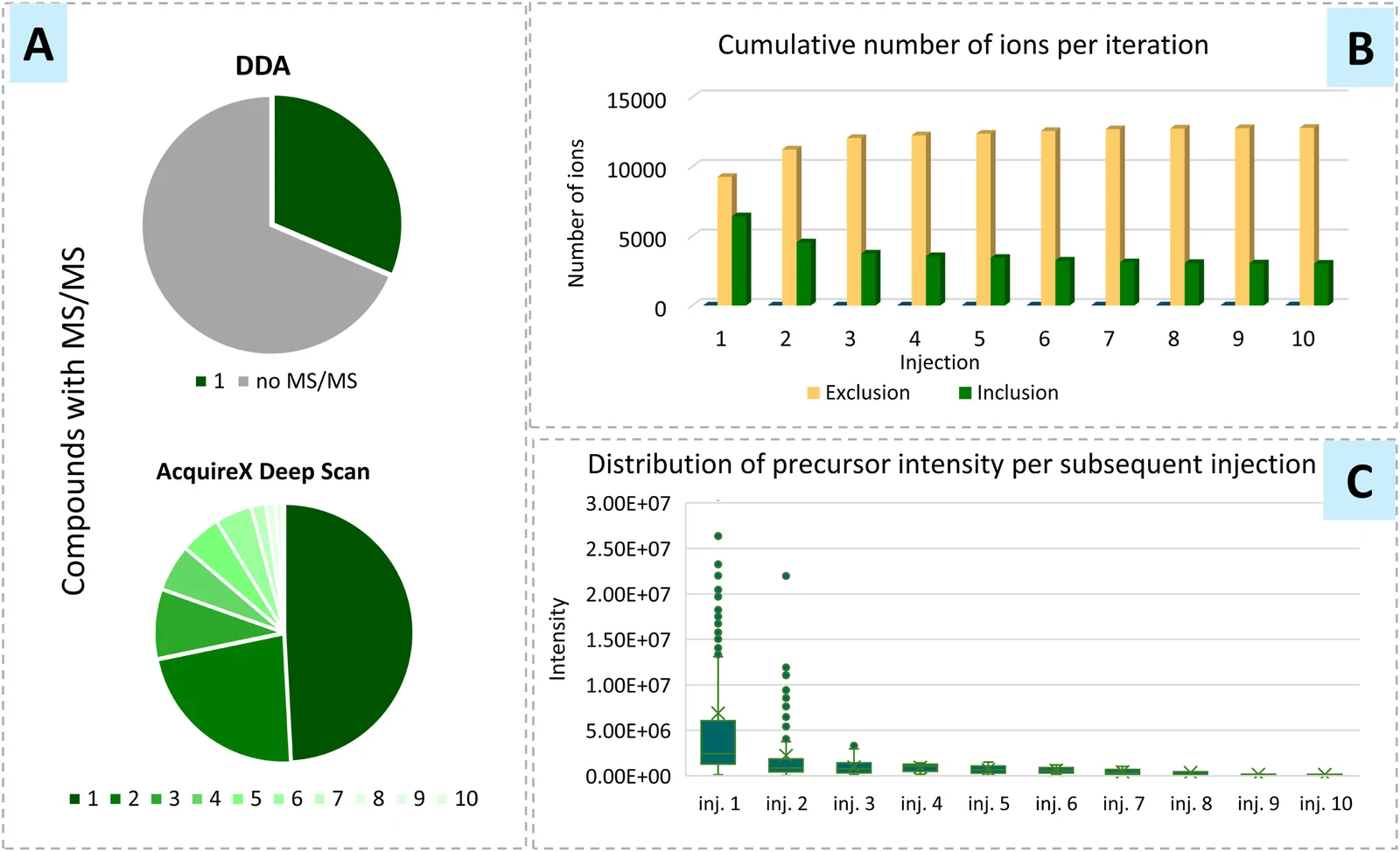
**Performance of AcquireX™ deep scan workflow**. (A) Number of compounds for which MS/MS spectra were acquired using standard DDA compared with AcquireX™ workflow. (B) Bar graph showing the number of ions (y-axis) included in the inclusion list (green) and exclusion list (yellow) automatically generated during AcquireX™ workflow. (C) Evolution of precursor-ion intensities across successive iterative injections in the AcquireX™ workflow.

AcquireX™ fragmented substantially more precursor ions than standard DDA (Fig. 1A). While DDA targets only the most intense ions above a fixed threshold, AcquireX™ progressively targets additional ions over successive injections. Moreover, the workflow continuously increased MS/MS coverage by iteratively refining inclusion and exclusion lists, a behavior consistent with previous studies (Razafindrabenja et al., 2025; Yu et al., 2022). Abundant ions were fragmented first and then excluded, allowing the algorithm to target less intense features in subsequent runs. This is reflected by the progressive increase in excluded ions and the parallel decrease in inclusion-list entries across iterations (Fig. 1B). The decrease of precursor intensities over iterations in Fig. 1C reflects this shift from high-to low-abundance ions, indicating reduced redundant fragmentation.

**Fig. 2:**
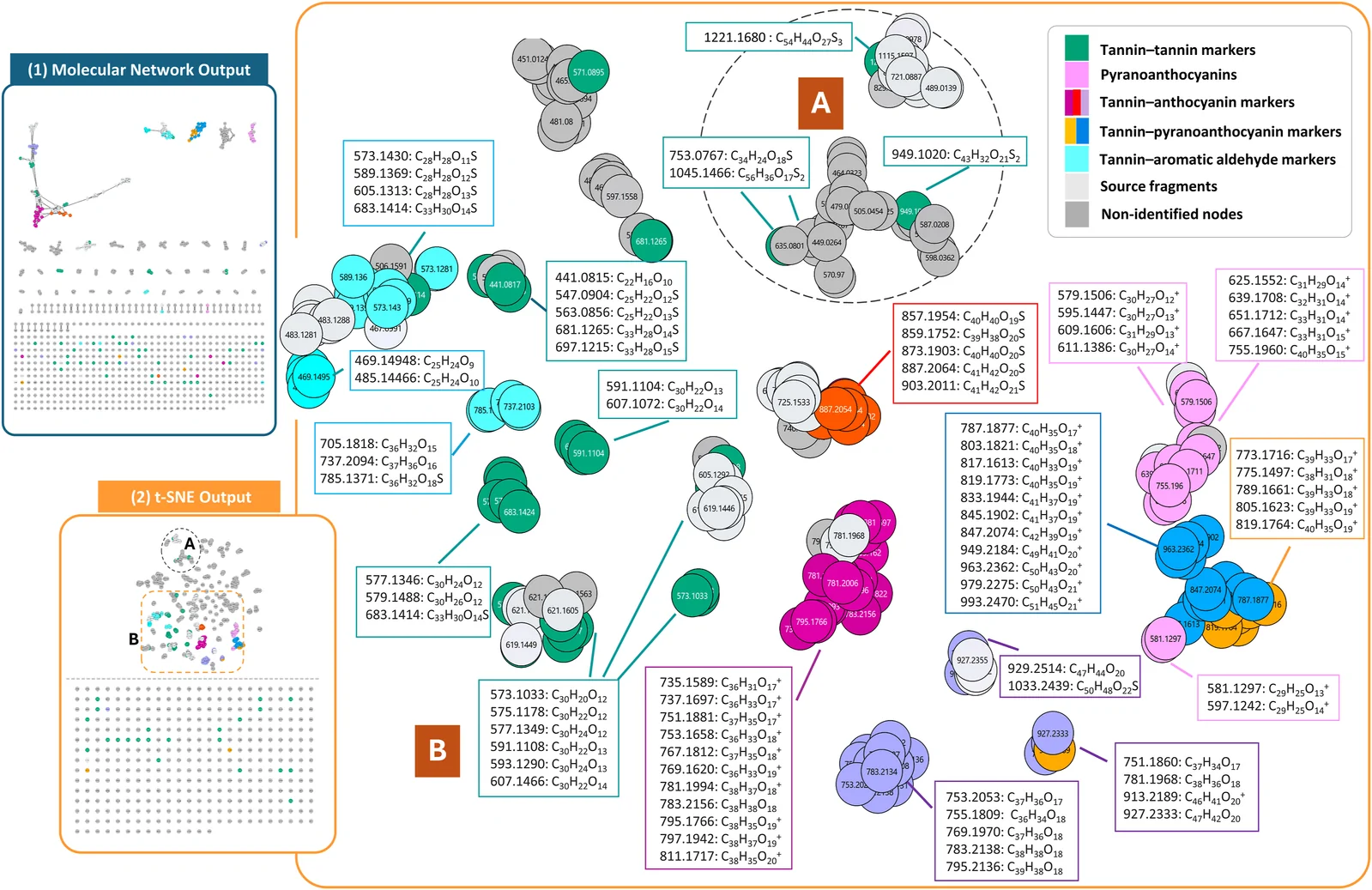
MN (1) and t-SNE (2) outputs for depolymerized tannin fractions. The nodes are labelled by the precursor ion *m/z* value.

MS/MS data were used to construct a MN, visualizing the structural relationships among detected features. To our knowledge, this is the first correlation-based MN generated from a depolymerized wine tannin fraction. The MzMine-preprocessed dataset was subsequently processed with Metgem to overcome inherent limitations to the GNPS workflow. Fig. 2 displays the resulting MN, shown in two complementary windows. The first provides a GNPS-style MN representation, clearly separating clusters and highlighting the diversity of evolution markers, with 921 nodes, 636 of which are connected into 24 clusters (cosine score ≥ 0.7) for precursor ions with *m/z* > 440 to focus on relevant markers. The second window presents the projection obtained using the t-distributed Stochastic Neighbor Embedding (t-SNE) algorithm, which reduces the high-dimensional MS/MS similarity matrix into a two-dimensional space. This graph preserves local relationships between structurally related groups of spectra and complements the network view (Olivon et al., 2018; Elie et al., 2019). Each node represents a feature defined by accurate mass, retention time, and MS/MS spectrum. Due to this local similarity preservation principle of t-SNE, highly similar features may appear partially or completely overlapped in the two-dimensional projection, reflecting their close spectral and structural similarity.

The MN revealed that oxidation-related features are organized according to their chemical similarity, suggesting that compounds generated through related transformation pathways tend to group together within the network. A total of 139 markers were characterized and classified into four molecular families according to the type of chemical interaction involved: tannin-tannin oxidation products (green nodes), tannin-anthocyanin condensation markers and oxidation markers (red, purple and fuchsia nodes), tannin-pyranoanthocyanin interaction markers (orange and blue nodes), and tannin-aromatic aldehyde interaction markers (light blue nodes). Some fragment ions, directly connected to marker nodes, were particularly informative for structure proposal and are shown as light grey nodes in the network. Pyranoanthocyanins are highlighted in pink, while features that remain unidentified are shown in dark grey. The markers identified for each molecular family are presented in the following sections.

#### 3.1.3 Compound identification

MN analysis revealed several nodes corresponding to oxidation and non-oxidation products from tannin–tannin interactions and reactions with other wine constituents. Because CT evolution products are structurally diverse, often occur at low abundance, and are unavailable as authentic standards, their characterization relied on a combination of elemental compositions, unambiguously determined by accurate mass measurements using UHRMS Orbitrap, MS/MS fragmentation data, MN relationships, and comparison with previously published data on tannin oxidation markers.

**Fig. 3:**
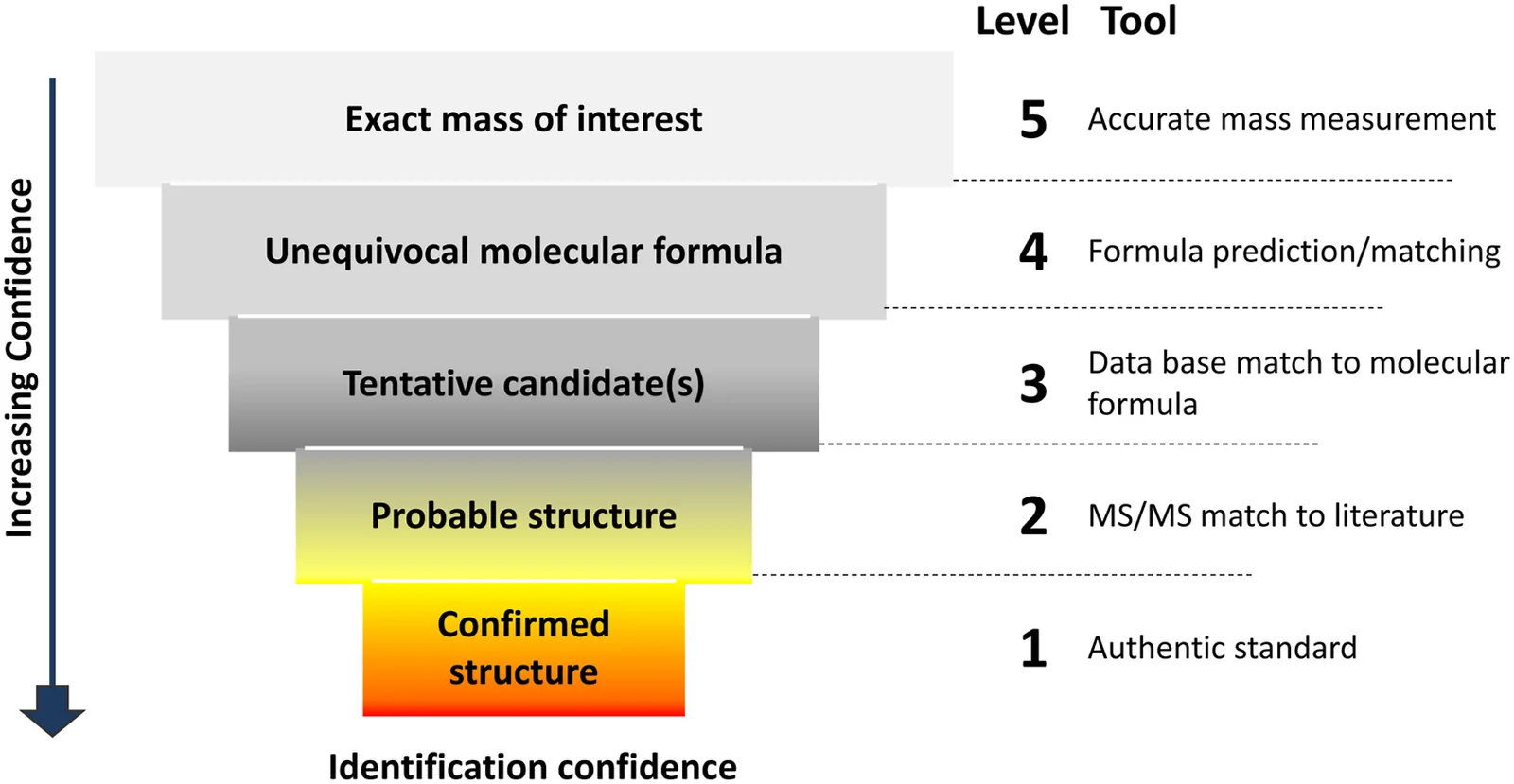
Schymanski confidence levels for categorizing the degree of uncertainty in the identification of small molecules by HRMS (adapted from Schymanski et al., 2014).

Schymanski et al. proposed a five-level confidence framework to improve the consistency and transparency of compound annotation in HRMS studies (Fig. 3). The system classifies annotations from level 1, corresponding to confirmed structures supported by reference standards, to level 5, representing features characterized only by their exact mass. Intermediate levels include level 2, probable structures supported by database or literature matching, level 3, tentative candidates where several isomeric possibilities may exist, and level 4, unambiguous molecular formulas (Schymanski et al. (2014)).

##### 3.1.3.1 Tannin*–*tannin markers

MN analysis revealed several nodes corresponding to oxidation products from tannin–tannin interactions (green nodes). 12 types of previously reported oxidation markers (M1–M12) were annotated at confidence levels 2–3 (probable structures or tentative candidates), each marker displaying up to 3 or 4 isomers, identified (Table 1). The presence of multiple isomers reflects that a single *m/z* value can correspond to different structures arising from different coupling positions and stereochemical configurations, which can be distinguished by different retention times.

**Table 1:** Known oxidation markers of tannin−tannin interactions (green nodes): retention time (UHPLC), minimum oxidation level and principal fragment ions from the MS/MS spectrum (3 major fragment ions in **bold,** major fragment ion underlined).

Marker	*m/z*	RT (min)	Molecular formula	*m/z* theoretical mass	*m/z* measured mass	Error (ppm)	Ox. Level^a^	MS/MS fragment ions
M1a	441	6.09	C_22_H_16_O_10_	441.08162	441.08133	−0.66	2	**441.0816**, **315.0500**, 303.0510, 287.0555, 255.0290, **139.0396**
M1b		8.92	C_22_H_16_O_10_	441.08162	441.08154	−0.18	2	**441.0815**, 331.0460, **315.0499**, 287.0569, 271.0600, 179.0341, **1,530,183**, 139.0389
M1c		12.29	C_22_H_16_O_10_	441.08162	441.08174	0.27	2	441.0817, **331.0449**, 315.0498, 287.0548, **271.0599**, **179.0338**, 153.0182
M2a	573	12.99	C_30_H_20_O_12_	573.10612	573.10328	−4.96	4	**573.1032**, 555.0952, 467.0960, **447.0744**, 387.0529, **373.0373**, 345.0425, 331.0816, 299.0549, 289.0704, 243.0318, 179.0338
M2b		12.27	C_30_H_20_O_12_	573.10612	573.10332	−4.89	4	**573.1025**, 555.0950, 467.0981, **447.0743, 421.0940**, 387.0549, 373.0376, 345.0433, 331.0820, 313.0715, 299.0556, 287.0553, 269.0452, 243.0319
M2c		11.02	C_30_H_20_O_12_	573.10612	573.10514	−1.71	4	**573.1061**, 555.0934, **447.0747**, 421.0915, **373.0374**, 345.0430, 297.0756, 243.0319, 153.0184, 123.0443
M3	575	12.18	C_30_H_22_O_12_	575.1184	575.11782	−1.01	3	**575.1177**, 557.1082, 469.1098, 449.0868, 425.0856, 421.0930, 407.0747, 377.0656, 329.0662, **299.0555**, 287.0547, **259.0603**, 163.0391, 123.0443
M4a	577	5.15	C_30_H_24_O_12_	577.13405	577.13399	−0.10	2	577.1378, **559.1250**, 541.0355, **425.0867**, 409.0929, 407.0777, 391.0826, 299.0558, 287.0561, 271.0608, 259.0609, 257.0457, 139.0391, **123.0446**
M4b		6.55	C_30_H_24_O_12_	577.13405	577.13456	0.88	2	**577.1365**, 559.1255, 541.1136, **425.0880**, **409.0921**, 259.0614, 257.0452, 139.0394, 123.0446
M4c		8.34	C_30_H_24_O_12_	577.13405	577.13461	0.97	2	577.1378, **559.1245**, 541.1142, **425.0867**, **409.0920**, 407.0775, 389.0654, 299.0551, 287.0559, 271.0612, 259.0610, 257.0442, 139.0390, 123.0443
M4d		7.80	C_30_H_24_O_12_	577.13405	577.13486	1.40	2	**577.1362**, 437.0884, **425.0868**, 299.0560, **287.0549,** 247.0611, 163.0400, 123.0445
M5a	579	3.92	C_30_H_26_O_12_	579.1497	579.14883	−1.50	1	579.1519, 561.1397, 543.1300, 441.1179, 427.1023, 409.0919, 393.0984, 301.0715, 289.0707, 271.0605, **259.0600**, 247.0604, 163.0390, **139.0390**, **123.0440**
M5b		3.81	C_30_H_26_O_12_	579.1497	579.14912	−1.00	1	579.1530, 561.1412, 543.1314, **427.1039**, 409.0926, 393.0986, 373.7646, 289.0720, 287.0563, 271.0612, 259.0608, 247.0610, 163.0394, **139.0390**, **123.0445**
M6a	593	8.95	C_30_H_24_O_13_	593.12896	593.12866	−0.51	2	**593.1288**, 557.1105, **467.0980**, 453.0829, 441.0816, 423.0726, 327.0512, 315.0503, 303.0504, 287.0561, 275.0556, 139.0393, **123.0441**
M6b		6.10	C_30_H_24_O_13_	593.12896	593.129	0.07	2	**593.1299**, 575.1212, 557.1090, **467.0981**, 453.0832, **441.0819**, 423.0724, 315.0501, 303.0499, 287.0559, 139.0395, 123.0445
M7	681	12.51	C_33_H_28_O_14_S	681.12725	681.12649	−1.12	2	681.1239, 575.1186, **529.0798**, **455.0564**, 437.0894, 407.0767, 299.0560, **287.0549**, 271.0600, 153.0547, 139.0396, 123.0446
M8a	683	10.74	C_33_H_30_O_14_S	683.1429	683.14143	−2.15	1	683.1459, **577.1336**, 559.1224, 531.0952, 513.0857, 425.0868, 409.0913, 407.0762, 395.0795, 379.0826, 361.0391, **289.0706**, 271.0599, 247.0601, 227.0372, **163.0390**, 127.0390
M8b		8.94	C_33_H_30_O_14_S	683.1429	683.14195	−1.39	1	577.1344, 407.0764, 323.1398, **289.0705**, 271.0613, **139.0395**, **123.0445**
M8c		8.34	C_33_H_30_O_14_S	683.1429	683.14238	−0.76	1	683.1381, 577.1353, 559.1235, **425.0870**,**409.0932**, 407.0768, **299.0560**, 271.0607, 257.0455, 163.0396, 127.0394
M9	697	11.22	C_33_H_28_O_15_S	697.12217	697.12145	−1.03	2	697.1231, 591.1151, **545.0748**, 501.0162, **471.0925**, 429.0837, **303.0499**, 243.0322, 153.0188, 123.0446
M10	736	10.73	C_28_H_22_O_19_N_3_S	736.05627	736.05574	−0.72	1	**736.0534**, 648.0556, **630.0453**, **612.0347**, 594.0262, 504.0162, 482.0020, 464.0998, 343.9979
M11	787	10.74	C_36_H_34_O_16_S_2_	787.1361	787.13554	−0.71	1	787.1422, 681.1315, **575.1181**, **557.1096**, 529.0791, 449.0903, 407.0777, 299.0541, 271.0606, **139.0396**
M12	1221	10.74	C_54_H_44_O_27_S_3_	1221.16687	1221.16802	0.94	5	903.1460, **721.0891**, **615.0811**, 507.0612, **489.0496**, 433.0355, 363.0165, 345.0287, 327.0184

^a^Minimum oxidation level; RT: Retention time.

The annotated markers mainly consisted of dimers and trimers. Terminal-terminal units were detected at *m/z* 573.1033 (M2), 575.1178 (M3), 577.1346 (M4), 579.1488 (M5), and 593.1287 (M6). Mixed terminal–extension units were observed at *m/z* 681.1265 (M7), 683.1419 (M8), and 697.1215 (M9), the latter supported by one nucleophilic (Nu) adduct for dimers and up to two for trimers. Extension–extension units with nucleophilic adducts appeared at *m/z* 787.1355 (M11) and 1221.1680 (M12) (Mouls and Fulcrand, 2012, 2015; Deshaies et al., 2022; Razafindrabenja et al., 2025). Oxidation of tannins is associated with the formation of new covalent bonds resulting from oxidative coupling reactions. These linkages may occur either within the same macromolecule (intramolecular bonding) or between two separate macromolecules, typically involving A and B aromatic rings (intermolecular bonding) (Poncet-Legrand et al., 2010). As oxidation progresses, more successive bonds are formed, increasing the oxidation level and generating a series of structurally related compounds.

**Fig. 4:**
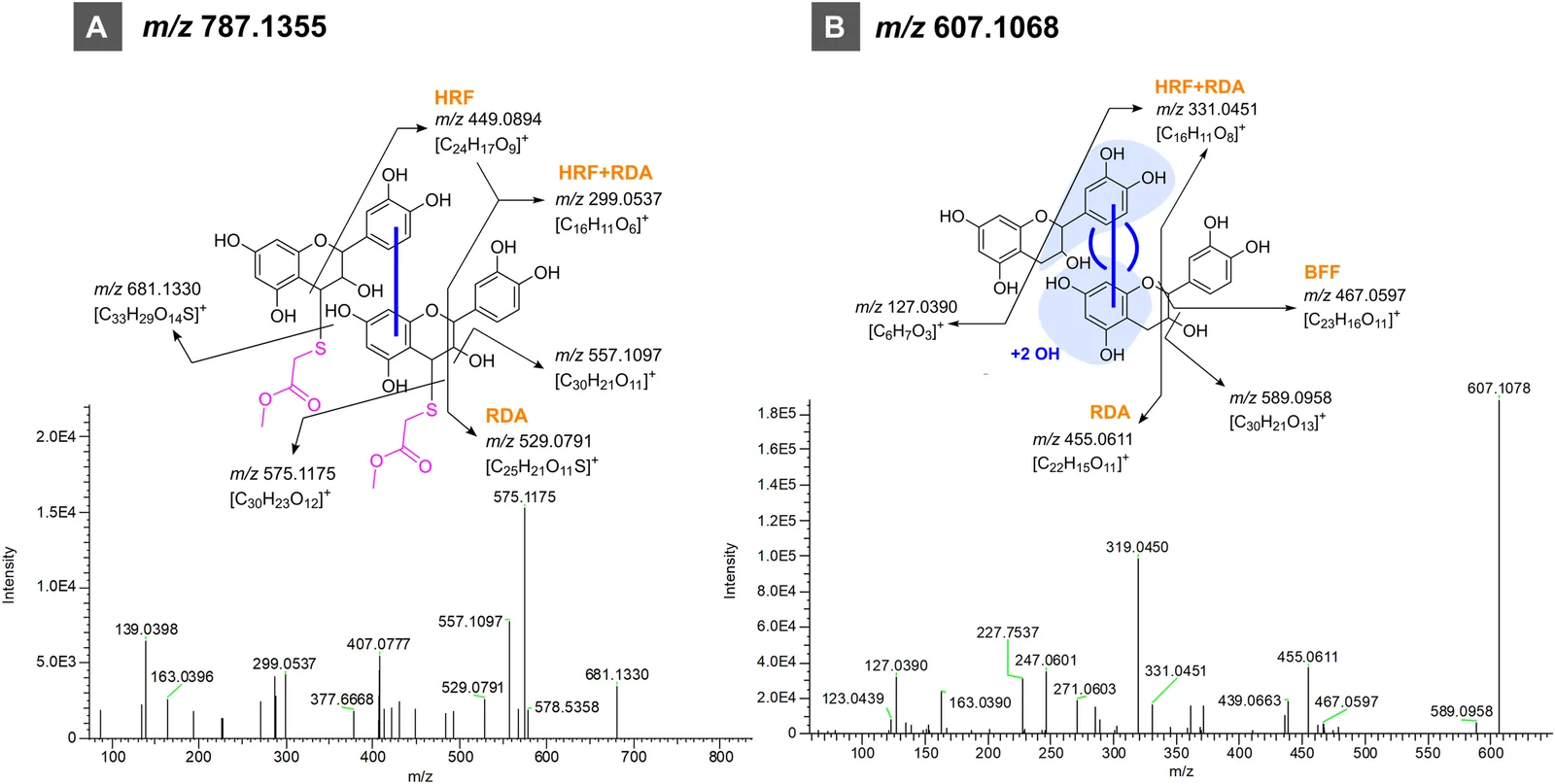
MS/MS spectrums and proposed fragmentation pathways of the oxidation markers at *m/z* 787.1355 (M11) (**A**) and at *m/z* 607.1068 (M16a) (**B**).

Successive mass differences of 2.015 Da indicate stepwise two hydrogen loss during oxidation, for example from *m/z* 579.1488 to 573.1033. The first oxidation level, observed at *m/z* 579.1488, corresponds to the formation of a single additional covalent bond. The second oxidation level, observed at *m/z* 577.1346, reflects further dehydrogenation and the formation of a second additional covalent bond, while the third level (with three additional covalent bonds) appears at *m/z* 573.1033. Additional hydroxylations were sometimes detected, which may reflect oxidative transformations, but could also arise from naturally occurring trihydroxylated flavan-3-ol units such as gallocatechin. Overall, considering isomers, the 23 known markers from 12 types displayed oxidation levels ranging from one to at least five, illustrating progressive tannin oxidation. All oxidation markers exhibited characteristic CT fragment ions in MS/MS spectra. Key fragmentation pathways included Retro-Diels-Alder cleavage (RDA, neutral losses of 138.032 and/or 152.047 Da), BenzoFuran-Forming fission (BFF, 122.036 Da), Heterocyclic Ring Fission (HRF, 126.031 Da), and water loss (18.011 Da), providing the diagnostic evidence that supports their assignment to levels 2–3. As an example, for the dimeric oxidation marker at *m/z* 787.1355 (M11), fragment ions at *m/z* 681.1330 and 575.1175 resulted from successive losses of 106.0025 Da, attributed to a Nu adduct (methyl thioglycolate, C_3_H_6_O_2_S), followed by interflavan linkage (IFL) cleavage during depolymerization reaction. RDA cleavage yields an ion at *m/z* 529.0791 (−152.053 Da), while the fragment at *m/z* 449.0894 corresponds to HRF cleavage (−126.028 Da) (Fig. 4A).

MN analysis also enabled the structural identification of 22 types of oxidation markers (M13–M34), corresponding to a total of 30 markers that, to the best of our knowledge, have not been previously reported (Table 2). Among these markers, based on the identification confidence scale (Schymanski et al., 2014), 13 new compounds (M13–M19) reached confidence levels 2–3 on the basis of clear diagnostic MS/MS evidence (characteristic RDA, HRF, BFF and/or Nu losses together with coherent network placement). The remaining 17 features (M20–M34) could only be assigned to levels 3–4: their accurate-mass formulae and systematic presence of HRF and RDA cleavages support their classification as oxidized tannin markers, yet the exact substitution pattern or linkage position remains ambiguous. These level-3–4 markers are therefore presented as tentative candidates. The first group mainly comprised dimers formed by terminal–terminal units, at a minimum oxidation level of two with three OH groups (*m/z* 625.1008, M17) and at level 3, with *m/z* 591.1104 (M15) and 607.1068 (M16), bearing one and two additional OH groups, respectively) (Table 2). For instance, the MS/MS spectrum of the dimeric oxidation marker at *m/z* 607.1068 (M16a), for which three isomers were detected, showed fragment ions at *m/z* 589.0958 (−18.011 Da, H_2_O loss), *m/z* 467.0597 (−122.036 Da, BFF), and *m/z* 455.0611 (−152.046 Da, RDA), suggesting that the additional OH groups are not located on the B-ring side of the second flavan-3-ol unit (Fig. 4B). However, the exact IFL position could not be unambiguously assigned, as several structural arrangements are possible, generating the observed isomers (M16a–M16d).

**Table 2:** New oxidation markers of tannin−tannin interactions (green nodes): retention time (UHPLC), minimum oxidation level and principal fragment ions from the MS/MS spectrum (3 major fragment ions in **bold,** major fragment ion underlined).

Marker	*m/z*	RT (min)	Molecular formula	*m/z* theoretical mass	*m/z* measured mass	Error (ppm)	Ox. Level^a^	MS/MS fragment ions
**New oxidation markers – confident identification level 2–3**								

M13	563	10.44	C_25_H_22_O_13_S	563.08539	563.08561	0.39	1	**563.0815**, 457.0783, 303.0512, **287.0548**, 243.0324, **153.0181**
M14	571	12.85	C_30_H_18_O_12_	571.0871	571.08948	4.17	5	**571.0872**, **465.0813**, 433.0550, 419.0433, 387.0181, 345.0605, 313.0340, **271.0233**
M15a	591	10.82	C_30_H_22_O_13_	591.11332	591.11041	−4.92	3	**591.1105**, 573.0973, 465.0800, 421.0549, 345.0604, 315.0502, **303.0499**, 301.0697, 289.0340, 285.0392, **271.0233**, 247.0599, 123.0440
M15b		12.01	C_30_H_22_O_13_	591.11332	591.11081	−4.25	3	**591.1101**, 573.1051, 563.1172, 465.0819, **441.0817**, 429.0832, 411.0719, 315.0513, **303.0501**, 275.0550, 163.0391, 133.0860
M15c		7.5	C_30_H_22_O_13_	591.11332	591.11263	−1.17	3	**591.1130**, 573.1038, 465.0834, 451.0678, **439.1001**, **411.0712**, 327.0509, 313.0351, 301.0350, 139.0396, 123.0445
M15d		8.15	C_30_H_22_O_13_	591.11332	591.11283	−0.83	3	**591.1131**, 573.1032, **439.0666**, 421.0560, 345.0614, 315.0504, **303.0501**, 285.0397, 271.0602, 247.0605, 163.0392, 127.0393
M16a	607	9.61	C_30_H_22_O_14_	607.10823	607.10682	−2.32	3	**607.1078**, 589.0956, 467.0611, **455.0608**, 439.0660, 437.0510, 331.0449, **319.0448**, 285.0393, 163.0390, 127.0390, 123.0440
M16b		5.98	C_30_H_22_O_14_	607.10823	607.10723	−1.65	3	**607.1075**, 589.0336, 467.0619, 455.0628, 439.0675, 361.0563, 331.0461, **319.0450**, 285.0402, 271.0602, **247.0602**, 163.0395, 127.0395
M16c		7.38	C_30_H_22_O_14_	607.10823	607.10742	−1.33	3	**607.1087**, 589.1003, 481.0768, 469.0760, 467.0622, 455.0627, **319.0451**, 301.0358, 271.0602, 247.0608, **163.0395**, 123.0446
M16d		12.09	C_30_H_22_O_14_	607.10823	607.10831	0.13	3	**607.1083**, **501.1004**, 483.0881, **383.0737**, 311.0540, 123.0443
M17	625	10.59	C_30_H_24_O_15_	625.11879	625.118	−1.26	2	625.1019, 607.0922, 519.0941, **473.0533**, 455.0450, 359.0241, **347.0217**, 333.0077, 303.0514, 287.0558, **139.0392**, 123.0446
M18	1223	10.73	C_54_H_46_O_27_S_3_	1223.14614	1223.14334	−2.29	4	903.1478, **721.0931**, **615.0836**, **489.0511**, 345.0293, 237.0080
M19	1239	8.6	C_54_H_46_O_28_S_3_	1239.14105	1239.14357	2.03	4	**829.0765**, **723.0690**, 631.0784, 541.0159, **505.0441**, 379.0122, 343.0135

**New oxidation markers – confident identification level 3–4**								

M20a	607	10.07	C_31_H_26_O_13_	607.14462	607.14656	3.19		**607.1472**, 589.1356, 571.1248, **481.1137**, 467.0975, 455.0626, 449.0878, 437.0880, 421.0929, 341.0667, 329.0663, **317.0658**, 289.0715, 139.0395, 123.0441
M20b		8.07	C_31_H_26_O_13_	607.14462	607.14731	4.43		**607.1487**, 589.1373, 571.1230, 501.1029, **481.1136**, 467.0986, 455.0980, 437.0878, 341.0667, 329.0661, 317.0661, **299.0559**, 289.0718, 139.0395, 123.0443
M21	655	12.98	C_28_H_30_O_14_S_2_	655.11497	655.11353	−2.20		**655.1499, 549.105**1, 503.0634, **443.0980**, 379.0486, 365.0668, 319.0274, 305.0664, 275.0551, 123.0444
M22	713	11.29	C_37_H_28_O_11_S_2_	713.11458	713.11794	4.71		**713.1169, 607.1094, 501.1004**, 455.0618, 383.0745, 353.0637, 123.0445
M23	735	12.98	C_36_H_30_O_15_S	735.13782	735.13462	−4.35		**735.1366**, 629.1276, 597.1011, 583.0899, 509.1071, **477.0316, 445.0558**, 431.0736, 399.0479, 357.0608, 355.0454, 325.0341, 283.0237, 123.0443
M24	751	10.85	C_34_H_22_O_18_S	751.05996	751.06197	2.68		751.1317, **645.0552**, 537.0369, 519.0265, **393.0133**, **375.0024**, 367.0141, 289.0710, 123.0445
M25	753	8.56	C_34_H_24_O_18_S	753.07561	753.07675	1.51		753.1668, 647.0720, 539.0498, **521.0856**, 503.0303, 413.0180, **395.0377**, 343.0125, 325.0040, 271.0127, 127.0393
M26a	839	12.82	C_39_H_34_O_17_S_2_	839.13102	839.1273	−4.43		**839.1287,** 733.1199, 627.1125, **595.0868**, 581.0740, **549.0473**, 507.0413, 489.0270, 465.0812, 433.0556, 312.9806, 271.0237, 123.0442
M26b		14.73	C_40_H_38_O_16_S_2_	839.16740	839.16528	−2.53		839.1650, **733.1234, 627.1494, 609.1401**, 501.1205, 483.1084, 351.0881, 339.0875, 283.0608, 243.0334, 139.0397
M27	845	10.74	C_32_H_28_O_25_S	845.07131	845.07175	0.52		845.0650, 739.0461, **557.0040, 450.9992**, 432.9903, **316.9955**, 300.9688, 139.0395
M28	853	8.18	C_41_H_40_O_20_	853.21857	853.22012	1.82		853.2222, 807.1479, **691.1657**, 619.1713, **539.1186**, 489.0839, 461.0913, **413.0891**, 313.0356, 123.0446
M29	895	3.85	C_35_H_42_O_27_	895.19862	895.20212	3.91		743.1577, **605.1240**, 587.1165, 467.0923, **453.0792**, 437.0840, 315.0453, 299.0504, **139.0389**
M30	909	3.85	C_43_H_40_O_20_S	909.19064	909.19009	−0.60		**619.1113**, 481.0820, **467.0653**, 347.0444, **329.0329**, 291.0875, 209.0126, 139.0392
M31	949	9.4	C_43_H_32_O_21_S_2_	949.09503	949.1002	5.45		949.1002, **843.0923**, 797.0552, 761.1262, 737.0868, 691.0481, 655.1074, 585.0360, 541.0160, 503.0658, 453.0158, **371.0442, 353.0335**, 325.0070, 237.0084
M32	951	10.93	C_35_H_34_O_27_S_2_	951.08016	951.08145	1.36		951.0815, **845.0712**, **739.0663**, **637.0700**, 531.0625, 505.0450, 387.0400, 369.0295
M33	1045	8.55	C_56_H_36_O_17_S_2_	1045.14667	1045.14657	−0.10		859.0903, 753.0798, 647.0736, **521.0404, 411.0747**, 395.0076, **287.0552**, 179.0347
M34	1269	8.54	C_55_H_48_O_29_S_3_	1269.15162	1269.15569	3.21		**753.0807, 647.0714**, 449.0314, **395.0077**, 343.0138

a Minimum oxidation level; RT: Retention time.

Two trimeric oxidation markers were also fully assigned at *m/z* 1223.1733 (M18) and 1239.1436 (M19), corresponding to an association of three extension units bearing three and four supplementary OH groups, respectively, and assigned to a minimum oxidation level of four. The MS/MS spectrum of the ion at *m/z* 1223.1733 showed fragment ion at *m/z* 615.0840, indicating successive losses of three Nu moieties (−318.007 Da) with rearrangement, followed by a neutral loss of flavan-3-ol unit (−288.081 Da). A high intensity fragment at *m/z* 489.0507 (HRF, −126.033 Da) further supported the proposed structure.

Although the remaining 17 markers (M20–M34) could not be fully identified by MS/MS alone, their systematic HRF (−126.03 Da) and RDA (−152.04 Da) cleavages support their assignment as oxidized tannin markers. Moreover, losses of 106.003 Da, observed for markers, including *m/z* 713.1179 (M22), 735.1346 (M23), and 949.1020 (M31) indicate the presence of extension units in their structures. Markers with higher *m/z* values, such as *m/z* 1239.1436, suggest more advanced oxidation levels and provide supplementary structural information that aligns with level 3 identification confidence (Table 2).

Interestingly, although some markers were distributed across multiple MN clusters (Fig. 2 (1)), t-SNE output provided a clearer representation by grouping structurally related features. Notably, the three isomeric oxidation markers at *m/z* 573.103 (M2a, M2b, and M2c), which appeared as self-looped nodes in MN, were grouped together in the t-SNE graph (Fig. 2 (2)).

##### 3.1.3.2 Tannin–anthocyanin markers

**Fig. 5:**
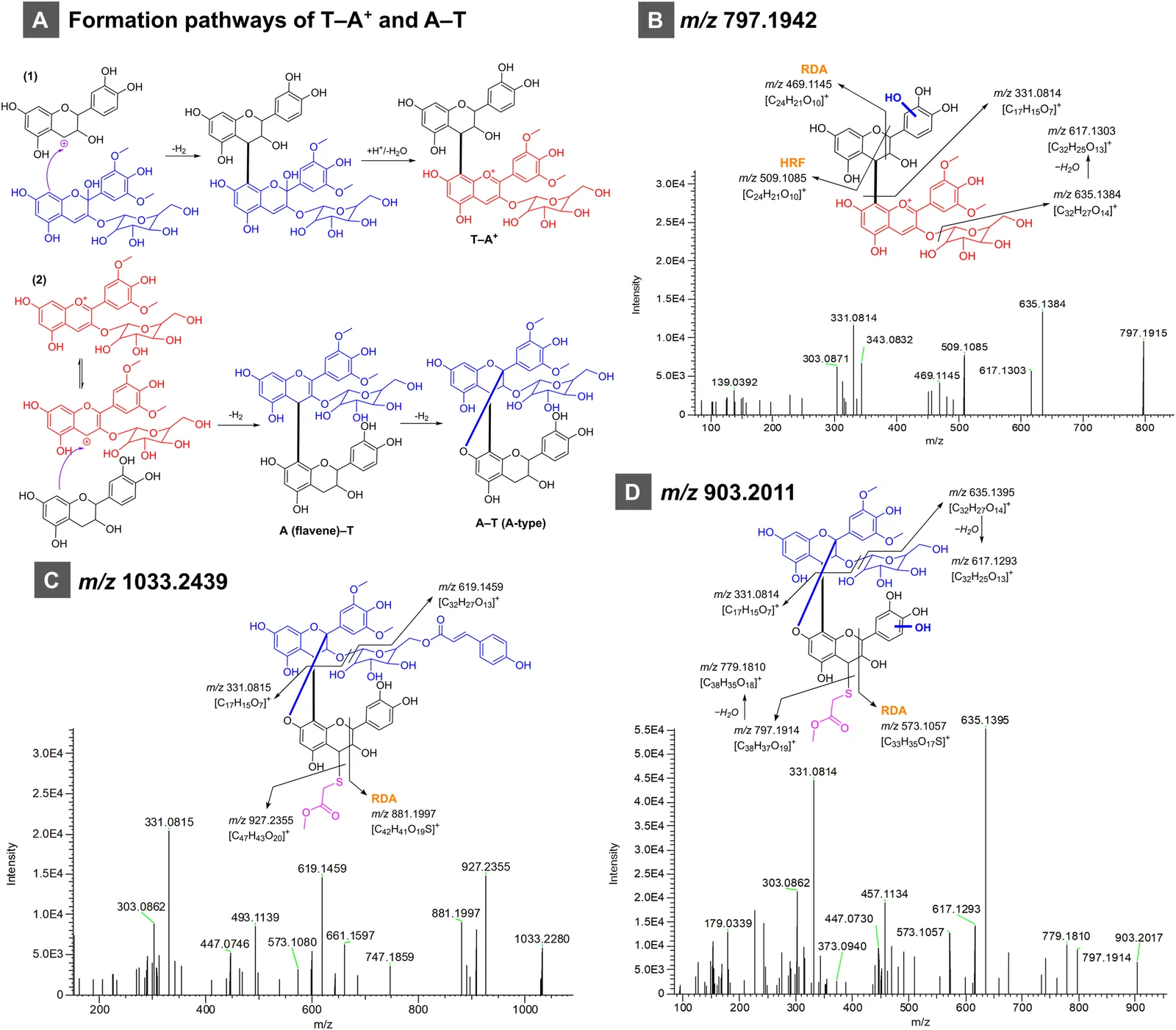
Formation mechanisms of T–A^+^ and A–T (**A**), MS/MS spectrums and proposed fragmentation pathways of the markers at *m/z* 797.1942 (M44) (**B**), 1033.2439 (M59) (**C**), and at *m/z* 903.2011 (M60) (**D**).

During red wine aging, tannins react with anthocyanins, to form specific evolution markers, highlighted in the MN as red, fuchsia, and purple nodes. Annotation of the t-SNE output revealed 11 types of tannin–anthocyanin markers (T–A^+^ and T–A) (M35–M45, fuchsia nodes), corresponding to 17 markers, assigned to confidence levels 2–3. These markers are consistent with mechanisms involving nucleophilic attack of the C_6_ or C_8_ positions of the hydrated hemiketal anthocyanin A-ring onto the C4 position of a CT extension unit after IFL cleavage and carbocation formation. This leads to the formation of B-type inter-unit linkages and the formation of polymerized pigments in which the anthocyanin constitutes the terminal unit in its flavylium form (A^+^), corresponding to (T–A^+^) adducts (Fig. 5A (1)).

Among these markers, 7 type (M35–M41) have been previously reported and have been associated with non-oxidative evolutions of polyphenolic compounds during wine aging (Cheynier et al., 2006; Fulcrand et al., 2006; Li and Duan, 2019; Salas et al., 2004; Zeng et al., 2016). They involve common wine anthocyanins linked to (+)-catechin or (−)-epicatechin units, since the latter are more abundant in aged wines. Their MS/MS spectra systematically showed neutral loss of glucose (−162.052 Da) from the anthocyanin unit, followed by a characteristic HRF cleavage (−126.033 Da), which supports the assignment of the tannin moiety as the upper unit (Salas et al., 2003) (Table 3). Besides these linkages, both labile T–A adducts and more stable A-type adducts may form at wine pH (Salas et al., 2004). A-type linkages resist to chemical depolymerization (Remy-Tanneau et al., 2003); accordingly, the marker at *m/z* 783.2156 (M41) within this group is tentatively assigned to a T–A adducts bearing an A-type linkage. Its MS/MS fragmentation is characterized by an intense ion at *m/z* 621.1605, resulting from the loss of the glucose moiety (−162.029 Da), and its proximity in the MN to the ion at *m/z* 781.1996 suggests structural similarity.

**Table 3:** Known and new oxidation markers of tannin−anthocyanin interactions (purple, red, and fuchsia nodes): retention time (UHPLC), minimum oxidation level and principal fragment ions from the MS/MS spectrum (3 major fragment ions in **bold,** major fragment ion underlined).

Marker	Annotation	*m/z*	RT (min)	Molecular formula	*m/z* theoretical mass	*m/z* measured mass	Error (ppm)	Ox. Level^a^	MS/MS fragment ions
**Tannin–anthocyanin markers – confident identification level 2–3**									

Known evolution markers									

M35	EC/C–Cy-3-*O*-glc (flavylium)	737	12.2	C_36_H_33_O_17_^+^	737.17123	737.1697	−2.07	0	**737.1697**, 719.1629, **575.1218**, 569.1306, 551.1190, 449.0907, 425.0881, 299.0559, **287.0551**, 259.0611, 179.0344, 163.0397, 153.0191
M36	EC/C–Pn-3-*O*-glc (flavylium)	751	12.62	C_37_H_35_O_17_^+^	751.18688	751.18808	1.60	0	751.1881, 733.1766, 715.1666, 631.1469, **589.1343**, 571.1252, 493.0777, 463.1016, 439.1026, 325.0718, 313.0708, **301.0705, 273.0756**, 163.0393, 153.0185
M37	EC/C–Dp-3-*O*-glc (flavylium)	753	11.77	C_36_H_33_O_18_^+^	753.16614	753.16578	−0.48	0	**753.1658**, 735.1557, 717.1479, 633.1257, **591.1129**, 585.1239, 573.1043, 465.0800, 441.0821, 315.0498, 303.0500, **275.0549**, 247.0601, 163.0391
M38a	EC/C–Pt-3-*O*-glc (flavylium)	767	12.28	C_37_H_35_O_18_^+^	767.18179	767.18125	−0.70	0	**767.1812**, 749.1709, 731.1612, 647.1390, 605.1288, 599.1407, 587.1174, 479.0967, 455.0975, 437.0881, 329.0659, **317.0655**, **289.0706**, 275.0557, 153.0182
M38b	EC/C–Pt-3-*O*-glc (flavylium)		9.15	C_37_H_35_O_18_^+^	767.18179	767.18221	0.55	0	**767.1822**, **605.1329**, 479.0990, 317.0670, **289.0721**, 153.0187
M39	EC/C–Dp-3-*O*-glc (flavylium)	769	10.63	C_36_H_33_O_19_^+^	769.16106	769.162	1.23	0	769.1620, 751.1527, 649.1229, **607.1081**, 589.0989, 441.0836, 429.0838, **303.0500**, **275.0555**, 247.0605, 179.0347, 153.0189
M40a	EC/C–Mv-3-*O*-glc (flavylium)	781	12.3	C_38_H_37_O_18_^+^	781.19744	781.19855	1.42	0	**781.1985, 619.1091**, 601.1367, 493.0768, 469.1151, 451.0676, 343.0829, **331.0814**, 303.0508, 289.0709, 271.0621, 153.0191, 123.0445
M40b	EC/C–Mv-3-*O*-glc (flavylium)		10.33	C_38_H_37_O_18_^+^	781.19744	781.19862	1.51	0	**781.1986, 619.1448**, 601.1356, 583.1238, 493.1152, 465.1196, 437.0882, 343.0829, **331.0812**, 303.0864, 287.0559, 271.0613, 247.0612, 123.0446
M40c	EC/C–Mv-3-*O*-glc (flavylium)		11.88	C_38_H_37_O_18_^+^	781.19744	781.19904	2.05	0	**781.1990, 619.1451**, 601.1358, 591.1515, 583.1259, 493.1128, 469.1136, 465.1203, 439.1041, 343.0826, **331.0812**, 303.0868, 289.0720, 271.0435, 247.0611, 123.0447
M40d	EC/C–Mv-3-*O*-glc (flavylium)		11.69	C_38_H_37_O_18_^+^	781.19744	781.19937	2.47	0	**781.1994, 619.1449**, 601.1351, 493.1130, 465.1205, 451.1041, 355.0832, **331.0813**, 303.0868, 289.0725, 275.0928, 271.0609, 247.0610, 153.0191, 139.0396, 123.0448
M40e	EC/C–Mv-3-*O*-glc (flavylium)		12.03	C_38_H_37_O_18_^+^	781.19744	781.19962	2.79	0	**781.1996, 619.1451**, 493.1146, 469.1137, 465.1207, 437.0893, 343.0824, **331.0813**, 303.0868, 289.0721, 271.0603, 247.0607, 153.0187, 123.0444
M40f	EC/C–Mv-3-*O*-glc (flavylium)		12.47	C_38_H_37_O_18_^+^	781.19744	781.20063	4.08	0	**781.2006, 619.1560**, 601.1428, 493.0749, 469.1135, 451.0690, 343.0808, **331.0812**, 303.0513, 289.0715, 271.0609, 247.0616, 153.0182, 139.0398, 123.0447
M41	EC/C–Mv-3-*O*-glc (A type)	783	10.13	C_38_H_38_O_18_	783.21309	783.21563	3.24	0	783.2156, **621.1606**, **469.1152**, 451.1047, 343.0827, 239.1565, 163.0394, **123.0448**

New oxidation/evolution markers									

M42	EC/C–Cy-3-*O*-glc (flavylium)	735	11.02	C_36_H_31_O_17_^+^	735.15558	735.15894	4.58	1	**735.1589**, 717.1502, **573.1058**, 555.0959, 537.0862, 481.0585, **447.0742**, 419.0806, 373.0379, 331.0817, 313.0716, 271.0276, 243.0323, 139.0393
M43	EC/C–Mv-3-*O*-glc (flavylium) + OH	795	12.82	C_38_H_35_O_19_^+^	795.17671	795.17659	−0.15	1	**795.1766, 633.1236**, 615.1131, 507.0921, 479.0602, 451.0666, 447.0717, 331.0447, **303.0500**, 275.0915, 137.0234, 127.0390, 123.0442
M44	EC/C–Mv-3-*O*-glc (flavylium) + OH	797	11.39	C_38_H_37_O_19_^+^	797.19236	797.19418	2.29	0	797.1942, **635.1398**, 617.1297, **509.1093**, 469.0294, 343.0832, **331.0816**, 303.0535, 123.0446
M45	EC/C–Mv-3-*O*-glc (flavylium) + 2OH	811	12.49	C_38_H_35_O_20_^+^	811.17162	811.17174	0.15	2	**811.1717, 649.0467**, 631.1082, 495.0935, 466.9714, 447.0728, 347.0407, 331.0457, **319.0448**, 303.0868

Anthocyanin–tannin markers									

Known oxidation markers									

M46	Pn-3-*O*-glc–EC/C (A type)	751	4.48	C_37_H_34_O_17_	751.18688	751.18602	−1.14	2	**751.1860**, **589.0842**, 571.1257, 437.0886, **343.0822**, 301.0718, 257.0451
M47a	Pn-3-*O*-glc–EC/C (A type)	753	10.87	C_37_H_36_O_17_	753.20253	753.20225	−0.37	1	753.2023, 673.1221, **591.1530**, 465.1190, **439.1046**, 411.0765, **313.0705**, 301.0715, 139.0399, 123.0448
M47b	Pn-3-*O*-glc–EC/C (A type)		9.24	C_37_H_36_O_17_	753.20253	753.20531	3.70	1	753.2053, **647.0831**, 601.1558, 591.1526, 475.1245, 465.1189, **439.1026**, 421.0935, **313.0707**, 301.0715, 123.0232
M48a	Dp-3-*O*-glc–EC/C (A type)	755	8.94	C_36_H_34_O_18_	755.18179	755.18081	−1.30	1	755.1808, 603.1361, **593.1289**, 467.0993, **441.0813**, 423.0715, 327.0509, **315.0497**, 303.0510, 139.0395
M48b	Dp-3-*O*-glc–EC/C (A type)		6.09	C_36_H_34_O_18_	755.18179	755.18086	−1.23	1	755.1809, **603.1341**, 593.1304, 575.1205, **467.0983**, 453.0835, **441.0817**, 423.0720, 327.0513, 315.0499, 303.0513, 139.0394
M49a	Pt-3-*O*-glc–EC/C (A type)	769	8.1	C_37_H_36_O_18_	769.19744	769.19704	−0.52	1	769.1970, 643.1670, **617.1504**, 607.1465, 481.1133, **455.0976**, 437.0886, **329.0421**, 317.0664, 123.0446
M49b	Pt-3-O-glc–EC/C (A type)		10.06	C_37_H_36_O_18_	769.19744	769.19875	1.70	1	769.1988, 617.1522, **607.1445**, 589.1370, 481.1151, **455.0974**, 437.0890, 341.0667, **329.0656**, 317.0668, 275.0555, 123.0446
M50	Mv-3-*O*-glc–EC/C (A type)	781	4.63	C_38_H_36_O_18_	781.19744	781.19685	−0.76	2	**781.1969**, **619.1449**, 601.1343, 479.0977, 465.1179, **373.0927**, 331.0275, 285.0404, 257.0452
M51a	Mv-3-*O*-glc–EC/C (A type)	783	9.27	C_38_H_38_O_18_	783.21309	783.21117	−2.45	1	**631.1690**, 621.1558, 495.1303, **469.1151, 343.0815**, 331.0823, 247.0611, 123.0445
M51b	Mv-3-*O*-glc–EC/C (A type)		6.73	C_38_H_38_O_18_	783.21309	783.21145	−2.09	1	783.2115, **631.1684**, 621.1607, 603.1542, 505.1354, 495.1303, **469.1142, 343.0813**, 307.2100
M51c	Mv-3-*O*-glc–EC/C (A type)		9.5	C_38_H_38_O_18_	783.21309	783.21232	−0.98	1	783.2123, **631.0967**, 621.1607, 603.1503, 505.1346, **495.0835, 469.1131**, 451.1027, 343.0429, 331.0580, 123.0441
M51d	Mv-3-O-glc–EC/C (A type)		8.87	C_38_H_38_O_18_	783.21309	783.21316	0.09	1	783.2132, 631.1678, 621.1560, 495.1305, **469.1135**, 451.1050, **343.0815**, **331.0815**, 315.0877, 127.0400
M51e	Mv-3-*O*-glc–EC/C (A type)		11.08	C_38_H_38_O_18_	783.21309	783.21342	0.42	1	783.2134, 631.1658, **621.0925**, 603.1500, 495.1286, **469.0497**, 451.1028, 355.0832, **343.0560**, 331.0812, 315.0865, 289.0716, 247.0614, 139.0396, 127.0393
M51f	Mv-3-*O*-glc–EC/C (A type)		9.05	C_38_H_38_O_18_	783.21309	783.2138	0.91	1	783.2138, 631.1676, **621.1609**, 495.1310, **469.1134**, 451.1050, 343.0816, **331.0814**, 303.0861, 123.0447
M52	Pt-3-*O*-glc–ethyl–EC/C (A type)	795	7.53	C_39_H_38_O_18_	795.21309	795.21361	0.65	1	**795.2136, 633.1613**, 507.1310, **481.1131**, 463.1033, 355.0208, 327.0510, 231.3074, 123.0445
M53	Pn-3-*O*-glc–EC/C–Nu (A type)	857	12.63	C_40_H_40_O_19_S	857.19573	857.19539	−0.39	1	857.1954, **751.1880**, 733.1802, **589.1336**, 543.0937, 463.1045, 417.0661, 313.0718, **301.0705**, 273.0763, 153.0190, 123.0445
M54	Dp-3-*O*-glc–EC/C–Nu (A type)	859	11.76	C_39_H_38_O_20_S	859.17499	859.17519	0.23	1	859.1752, **753.1658**, 735.1506, 697.1228, **591.1130**, 545.0764, 465.0839, 441.0835, 419.0447, 353.4972, **303.0501**, 275.0554, 139.0396
M55	Pt-3-*O*-glc–EC/C–Nu (A type)	873	12.27	C_40_H_40_O_20_S	873.19064	873.19027	−0.43	1	873.1903, **767.1814**, 749.1642, 721.1459, **605.1288**, 559.0926, 479.0992, 433.0599, 329.0664, **317.0655**, 289.0707, 163.0396, 153.0189, 123.0445
M56a	Mv-3-*O*-glc–EC/C–Nu (A type)	887	12.66	C_41_H_42_O_20_S	887.20629	887.20545	−0.95	1	887.2055, **781.1967**, 763.1861, 735.1597, 725.1545, 707.1468, **619.1446**, 601.1345, 573.1045, 493.1128, 469.1136, 447.0739, 343.0818, **331.0812**, 303.0862, 163.0391, 153.0186
M56b	Mv-3-*O*-glc–EC/C–Nu (A type)		12.29	C_41_H_42_O_20_S	887.20629	887.20638	0.10	1	**887.2064**, 781.0147, 763.1839, **619.1455**, 601.1351, 457.1153, **331.0819**, 303.0878, 163.0400, 153.0186
M57	Mv-3-*O*-(6-*O*-*p*-coumaroyl)-glc–EC/C (A type)	927	11.1	C_47_H_42_O_20_	927.23422	927.23328	−1.01	2	**927.2333**, 805.1379, **619.1448**, 601.1354, 467.0976, **373.0920**, 331.0823, 257.0454, 123.0445
M58	Mv-3-*O*-(6-*O*-*p*-coumaroyl)-glc–EC/C (A type)	929	12.64	C_47_H_44_O_20_	929.24987	929.2514	1.65	1	929.2514, **777.1992**, 759.1935, 615.1520, 495.1301, 469.1144, 451.1031, 427.0671, **343.0810**, 331.0813, **147.0441**
M59	Mv-3-*O*-(6-*O*-*p*-coumaroyl)-glc–EC/C–Nu (A type)	1033	13.49	C_50_H_48_O_22_S	1033.24307	1033.24391	0.81	1	1033.2439, 927.2348, 881.1992, 747.1513, **619.1456**, 573.1081, 493.1146, 343.0830, **331.0814**, 303.0869, **147.0332**

New oxidation markers									

M60	Mv-3-*O*-glc–EC/C–Nu + OH (A type)	903	12.26	C_41_H_42_O_21_S	903.20121	903.20111	−0.11	1	903.2011, 797.1931, 779.1826, 677.1494, **635.1394**, 617.1287, 573.1055, 457.1133, 435.0715, **331.0812**, **303.0520**, 243.0322, 179.0342, 153.0184, 123.0445

a Minimum oxidation level; EC/C: epicatechin/catechin; Dp: delphinidin; Cy: cyanidin; Pt: petunidin; Pn: peonidin; Mv: malvidin; glc: glucoside; Nu: nucleophile; RT: Retention time.

MN analysis also enabled the identification of new oxidized tannin–anthocyanin markers (fuchsia nodes) at *m/z* 735.1589, 795.1766, 797.1942, and 811.1717 (M42–M45), assigned to EC/C–Cy-3-*O*-glc and EC/C–Mv-3-*O*-glc structures, exhibiting at least one oxidation level, with one (M43, M44) or two additional OH groups (M45) (Table 3). The MS/MS spectrum of *m/z* 797.1942 showed fragment at *m/z* 635.1384 (−162.052 Da, glucose loss), followed by *m/z* 617.1303 (−18.008 Da, H_2_O loss). Further fragments at *m/z* 509.1085 and 469.1145, are consistent with HRF and RDA cleavages, respectively (Fig. 5B). Notably, the fragment at *m/z* 509.1085, corresponding to HRF cleavage, has an exact mass of C_26_H_21_O_11_, confirming that the anthocyanin unit is located at the upper position.

A distinct group of markers was attributed to anthocyanin–tannin reactions (A–T) in which the tannin moiety reacts as a nucleophile toward the electrophilic anthocyanin flavylium ion (purple and red nodes). These interactions may lead to the formation of intermediate anthocyanin (flavene)-tannin adducts, which can subsequently form biphenyl ether linkages between tannin and anthocyanin, yielding A-type derivative markers that are particularly resistant to acid-catalyzed cleavage (Fulcrand et al., 2006) (Fig. 5A (2)). 14 types of markers (M46–M59) (Table 3), corresponding to 23 evolution markers, previously reported, were highlighted (Remy-Tanneau et al., 2003; Sánchez-Ilárduya et al., 2014; Zeng et al., 2016). Interestingly, markers containing extension units (purple nodes) were distinguished from terminal units (red nodes). For example, the ion at *m/z* 783.2123 (M51c, Mv-3-*O*-glc–EC/C) produced an RDA fragment at *m/z* 631.1657. A fragment ion at *m/z* 621.1608, corresponding to glucose loss (−162.052 Da), was also observed. The combination of these two losses resulted in a fragment at *m/z* 469.1131, and these losses followed by HRF cleavage of the anthocyanin unit can be attributed to intense fragment at *m/z* 343.0813. Furthermore, markers at *m/z* 927.2332, 929.2513, and 1033.2439 (M57–M59) were characterized by a *p*-coumaroylated glucose moiety. While *p*-coumaroylated anthocyanins have been reported in wines, their association with tannin terminal or extension units has not been previously reported. In the present markers, the neutral loss of 308 Da (−C_15_H_16_O_7_) corresponds to the loss of the *p*-coumaroylated glucose (Fig. 5C).

Finally, a new oxidation marker at *m/z* 903.2011 (M60) was identified as Mv-3-*O*-glc–EC/C–Nu with an additional OH group. It MS/MS spectrum displayed successive losses of Nu (−106.009 Da, *m/z* 797.1914), water (−18.010 Da, *m/z* 779.1810), followed by glucose loss (−162.052 Da, *m/z* 635.1395) and RDA fragment at *m/z* 573.1057 (Table 3, Fig. 5D).

##### 3.1.3.3 Tannin–pyranoanthocyanin markers

MN analysis revealed a distinct family of pyranoanthocyanin–tannin markers, comprising 18 types of markers (M61–M78), corresponding to a total of 21 markers (Table 4) assigned to confidence levels 2–3, and distributed across orange, and blue nodes. These markers clustered in close proximity to a group of known pyranoanthocyanins, suggesting shared structural features and similar MS/MS fragmentation patterns. In the present dataset, 10 known pyranoanthocyanins (P1–P10) were annotated and clustered as pink nodes in MN. Their presence in the network helped to identify the neighboring pyranoanthocyanin–tannin markers. During aging, pyranoanthocyanins are formed through cycloaddition reactions at the C4 position of the flavylium form of anthocyanins with nucleophilic wine constituents such as vinylphenols, vinylcatechols, or acetaldehyde-derived intermediates formed during oxidation (Timberlake and Bridle, 1976; Fulcrand et al., 1996). Their MS/MS spectra systematically exhibited diagnostic neutral losses corresponding to the cleavage of glycosylated substituents, including glucose (−162.052 Da), 6-*O*-acetylglucose (−204.064 Da), and 6-*O*-*p*-coumaroylglucose (−308.089 Da). Notably, most of these identified compounds correspond to Pinotin A-related structures, the most abundant pyranoanthocyanins in aged red wines, whose concentration increases during bottle aging (Schwarz et al., 2004; Sun et al., 2020).

**Table 4:** Known and new markers of tannin–pyranoanthocyanin (orange and blue nodes) and tannin–aromatic aldehyde interactions (light blue nodes): retention time (UHPLC), minimum oxidation level and principal fragment ions from the MS/MS spectrum (3 major fragment ions in **bold,** major fragment ion underlined).

Marker	Annotation	*m/z*	RT (min)	Molecular formula	*m/z* theoretical mass	*m/z* measured mass	Error (ppm)	Ox. Level^a^	MS/MS fragment ions
**Pyranoanthocyanins**									

Known evolution markers									

P1	PyranoPn-3-*O*-glc-4-vinylphenol	579	12.6	C_30_H_27_O_12_^+^	579.1497	579.15056	1.48	0	**579.1506, 417.0969**, 279.0862, 251.0912, **163.0385**
P2	PyranoDp-3-*O*-glc-4-vinylphenol	581	11.68	C_29_H_25_O_13_^+^	581.12897	581.12969	1.24	0	**581.1296, 419.0763, 401.1233**, 383.1140, 355.1183, 181.0496, 163.0408, 123.0444
P3	PyranoPt-3-*O*-glc-4-vinylphenol	595	12.28	C_30_H_27_O_13_^+^	595.14462	595.14467	0.09	0	**595.1447, 433.0140, 418.0683**, 163.0386, 123.0440
P4	PyranoDp-3-*O*-glc-4-vinylcathecol	597	11.13	C_29_H_25_O_14_^+^	597.12388	597.12419	0.52	0	**597.1242, 435.0157**, 337.1702, 123.0444
P5	PyranoMv-3-*O*-glc-4-vinylphenol	609	12.68	C_31_H_29_O_13_^+^	609.16027	609.16011	−0.26	0	**609.1601, 447.0043**, 431.0384
P6	PyranoPt-3-*O*-glc-4-vinylcathecol	611	11.97	C_30_H_27_O_14_^+^	611.13953	611.13856	−1.59	0	**611.1395, 449.0868**, 163.0394, 123.0445
P7	PyranoMv-3-*O*-glc-4-vinylcathecol (Pinotin A)	625	12.42	C_31_H_29_O_14_^+^	625.15518	625.15519	0.01	0	**625.1552, 463.0169**, 447.0304
P8	PyranoMv-3-*O*-(6-*O*-*p*-acetyl)-glc-4-vinylphenol	651	12.97	C_33_H_31_O_14_^+^	651.17083	651.17115	0.49	0	651.1712, 447.1078, 431.0411, 163.0389
P9	PyranoMv-3-*O*-(6-*O*-*p*-acetyl)-glc-4-vinylcathecol	667	12.69	C_33_H_31_O_15_^+^	667.16575	667.16467	−1.61	0	**667.1647, 463.0668**, 447.0723, 163.0395
P10	PyranoMv-3-*O*-(6-*O*-*p*-coumaroyl)-glc-4-vinylphenol	755	13.29	C_40_H_35_O_15_^+^	755.19705	755.19603	−1.35	0	**755.1960, 447.0716**, 431.0764, 123.0442

**Pyranoanthocyanin–tannin markers – confident identification level 2–3**									

Known evolution markers									

M61	EC/C–PyranoMv-3-*O*-glc-4-vinylcatechol	913	9.75	C_46_H_41_O_20_^+^	913.21857	913.21892	0.38	0	**913.2189, 751.1650**, 733.1560, 599.1183, 571.1234, **505.1133**, 463.1039, 247.0604

New oxidation markers									

M62	PyranoPn-3-*O*-glc–EC/C	773	11.37	C_39_H_33_O_17_^+^	773.17123	773.17161	0.50	1	**773.1716, 611.1182**, 499.1050, **487.0667**, 459.0743, 403.0464, 287.0558, 127.0398
M63	PyranoDp-3-*O*-glc–EC/C	775	9.62	C_38_H_31_O_18_^+^	775.15049	775.1497	−1.02	1	**775.1497, 613.0335**, 543.0905, 503.0614, **501.0819**, 299.0590, 123.0444
M64a	PyranoPt-3-*O*-glc–EC/C	789	11.01	C_39_H_33_O_18_^+^	789.16614	789.1661	−0.05	1	**789.1661, 627.1130**, 517.0763, **515.0976**, 473.0868, 123.0443
M64b	PyranoPt-3-*O*-glc–EC/C		10.34	C_39_H_33_O_18_^+^	789.16614	789.16622	0.10	1	**789.1662, 627.1131**, 557.1092, 515.0976, **503.0611**, 459.0712
M65	PyranoPt-3-*O*-glc–EC/C + OH	805	9.19	C_39_H_33_O_19_^+^	805.16106	805.16234	1.60	1	**805.1623, 643.1083**, 601.0963, 531.0926, **503.0623**, 459.0709, 433.0560
M66	PyranoMv-3-*O*-glc–EC/C + OH	819	10.6	C_40_H_35_O_19_^+^	819.17671	819.17728	0.70	1	**819.1773, 657.0675**, 587.1207, **545.1082**, 531.0921, 123.0445
M67	PyranoMv-3-*O*-glc–EC/C + 2OH	835	11.72	C_41_H_39_O_19_^+^	835.20801	835.20609	−2.30	2	**835.2061, 673.1471**, 561.1309, **533.0984**, 123.0447

Portisins									

Known evolution markers									

M68	VinylpyranoCy-3-*O*-glc–EC/C	787	12.2	C_40_H_35_O_17_^+^	787.18775	787.18775	1.11	0	**787.1878, 625.1335,** 513.1179, **501.0817**, 457.0930, 123.0445
M69a	VinylpyranoDp-3-*O*-glc–EC/C	803	11.94	C_40_H_35_O_18_^+^	803.1821	803.1821	0.39	0	**803.1821, 641.0602**, 531.0920, **529.1130**, 123.0443
M69b	VinylpyranoDp-3-*O*-glc–EC/C		11.62	C_40_H_35_O_18_^+^	803.18236	803.18236	0.71	0	**803.1824, 641.1290**, 529.1130, **517.0767**, 473.0864, 405.0610, 123.0443
M70	VinylpyranoDp-3-*O*-(6-*O-p*-acetyl)-glc–EC/C	845	12.37	C_42_H_37_O_19_^+^	845.19236	845.19016	−2.60	0	**845.1902, 641.1288**, 531.0939, **529.1144**, 503.0979, 163.0398, 123.0441
M71	VinylpyranoMv-3-*O*-glc–EC/C	847	12.19	C_42_H_39_O_19_^+^	847.20741	847.20741	−0.70	0	**847.2074, 685.1544**, 657.1592, 573.1384, **531.0920**, 503.0972, 487.0637, 447.0711
M72	VinylpyranoDp-3-*O*-(6-*O-p*-coumaroyl)-glc–EC/C	949	12.66	C_49_H_41_O_20_^+^	949.21844	949.21844	−0.14	0	**949.2184, 641.1288**, 599.1205, 531.0923, **529.1122**
M73	VinylpyranoPt-3-*O*-(6-*O-p*-coumaroyl)-glc–EC/C	963	12.85	C_50_H_43_O_20_^+^	963.2362	963.2362	2.06	0	**963.2362**, **655.069**8, 543.1286, **531.0924**, 487.1041, 147.0444

New oxidation markers									

M74	VinylpyranoDp-3-*O*-glc–EC/C + OH	817	11.3	C_40_H_33_O_19_^+^	817.16133	817.16133	0.34	2	**817.1613**, 799.1483, **655.1082**, 627.1152, 543.0925, **529.0780**
M75	VinylpyranoDp-3-*O*-glc–EC/C + OH	819	10.44	C_40_H_35_O_19_^+^	819.17642	819.17642	−0.35	1	**819.1764, 657.1237**, 587.1158, **545.1086**, 517.0773, 413.0728, 273.0905, 123.0447
M76a	VinylpyranoPt-3-*O*-glc–EC/C + OH	833	11.72	C_41_H_37_O_19_^+^	833.1935	833.1935	1.37	1	**833.1935, 671.1399**, 559.0674, **531.0925**, 447.0716, 123.0442
M76b	VinylpyranoPt-3-*O*-glc–EC/C + OH		11.43	C_41_H_37_O_19_^+^	833.19443	833.19443	2.49	1	**833.1944, 671.0658**, 559.1247, **517.07**63, 473.0869, 289.0692, 123.0444
M77	VinylpyranoPt-3-*O*-(6-*O-p*-coumaroyl)-glc–EC/C + OH	979	12.6	C_50_H_43_O_21_^+^	979.22745	979.22745	−1.72	1	**979.2274, 671.1393**, 601.1335, **559.1236**, 531.0919, 123.0444
M78	VinylpyranoMv-3-*O*-(6-*O-p*-coumaroyl)-glc–EC/C + OH	993	12.83	C_51_H_45_O_21_^+^	993.24696	993.24696	2.19	1	**993.2470, 685.0906**, 573.1401, **531.0923**, 487.1022, 471.0736, 331.0828, 147.0442

Tannin–aromatic aldehyde markers									

New evolution markers – confident identification level 2–3									

M79	Coniferaldehyde–EC/C	469	10.35	C_25_H_24_O_9_	469.14948	469.14948	0.36	0	469.1495, 437.1238, 343.0816, 317.0667, **291.0864, 179.0346, 137.0534**, 123.0440
M80a	Coniferaldehyde–EC/C + OH	485	9.4	C_25_H_24_O_10_	485.14466	485.14466	0.90	0	485.1447, 467.1413, 333.0983, 301.0711, **291.0862, 195.0650, 153.0546**, 139.0390, 123.0440
M80b	Coniferaldehyde–EC/C + OH		10.27	C_25_H_24_O_10_	485.14467	485.14467	0.92	0	**485.1447**, 301.0718, **291.0867**, 195.0656, **153.0546**, 139.0392, 123.0446
M80c	Coniferaldehyde–EC/C + OH		8.91	C_25_H_24_O_10_	485.14472	485.14472	1.02	0	485.1447, 467.0275, 313.0720, **291.0863**, 289.0718, 273.0765, 195.0541, **153.0188, 139.0390**, 123.0324
M81a	Coniferaldehyde–EC/C–Nu	573	9.76	C_24_H_28_O_14_S	573.12809	573.12809	1.46	0	573.1281, 467.0938, 449.0843, 341.0661, **305.0441, 287.0550**, 179.0339, 151.0390, **127.0390**
M81b	Coniferaldehyde–EC/C–Nu		13.92	C_28_H_28_O_11_S	573.14302	573.14302	0.89	0	**573.1430**, 467.0961, 435.1089, 395.0805, 289.0708, 271.0604, **179.0347**, 163.0392, **137.0239**, 127.0390
M82a	Coniferaldehyde–EC/C–Nu + OH	589	14.38	C_28_H_28_O_12_S	589.13576	589.13576	−2.82	1	**589.1358, 483.0374, 451.1007**, 423.1060, 333.0958, 177.1039, 133.0862
M82b	Coniferaldehyde–EC/C–Nu + OH		13.26	C_28_H_28_O_12_S	589.13596	589.13596	−2.48	1	**589.1360, 483.0824**, 409.0869, 333.0952, 305.0660, 289.0716, 153.0186, **137.0597**, 123.0444
M82c	Coniferaldehyde–EC/C–Nu + OH		13.43	C_28_H_28_O_12_S	589.13692	589.13692	−0.86	1	589.1369, **483.0774**, 451.1031, 301.0714, 289.0705, 271.0597, 195.0651, **153.0188**, 137.0242, 127.0389, 123.0446
M82d	Coniferaldehyde–EC/C–Nu + OH		12.33	C_28_H_28_O_12_S	589.13768	589.13768	0.43	1	589.1377, **483.0620**, 451.1026, 433.0919, 395.0795, 391.0830, 323.0565, **289.0500**, 271.0599, 195.0651, **153.0188**, 127.0390, 123.0440
M82e	Coniferaldehyde–EC/C–Nu + OH		12.45	C_28_H_28_O_12_S	589.13787	589.13787	0.76	1	589.1379, 499.0700, **483.0898**, 451.1035, 433.0935, 395.0801, 391.0825, 323.0222, **289.0706**, 271.0600, 195.0651, **153.0184**, 127.0389, 123.0441
M82f	Coniferaldehyde–EC/C–Nu + OH		11.92	C_28_H_28_O_12_S	589.13954	589.13954	3.59	1	589.1395, **483.0913**, 465.1196, 411.0777, 347.0821, **305.0656**, 287.0560, 273.0759, 179.0339, 151.0393, **137.0233**, 122.9782
M82g	Coniferaldehyde–EC/C–Nu + OH		11.72	C_28_H_28_O_12_S	589.13979	589.13979	4.02	1	589.1398, 483.0898, 465.1197, 411.0754, 344.3668, 339.0515, **305.0655**, 287.0553, 273.0774, **179.0338**, **137.0598**, 127.0390, 123.0445
M83a	Coniferaldehyde–EC/C–Nu + 2OH	605	11.05	C_28_H_28_O_13_S	605.13133	605.13133	−1.67	1	**605.1313**, 499.1229, 481.1144, 453.0829, 425.0883, 411.0760, 317.0673, **305.0656**, 287.0549, 195.0651, 179.0339, **153.0546**, 127.0389
M83b	Coniferaldehyde–EC/C–Nu + 2OH		11.48	C_28_H_28_O_13_S	605.13156	605.13156	−1.29	1	605.1316, **499.1230**, 425.0863, 411.0764, 359.0766, 315.0912, **305.0653**, 287.0552, 195.0650, 179.0341, **153.0546**, 127.0389, 123.0444
M84	EC/C–hydroxymethylfurfural–EC/C	705	9.17	C_36_H_32_O_15_	705.18184	705.18184	0.63	0	**705.1818**, 553.1344, 535.1248, 427.1041, 413.0868, 401.0878, 383.0768, **355.0814**, 289.0705, **247.0602**, 163.0391, 127.0390
M85a	EC/C–aromatic aldehyde–EC/C	737	11.17	C_37_H_36_O_16_	737.20937	737.20937	2.39	0	737.2094, 585.1609, 567.1520, 507.1289, **449.1450**, 417.1191, 355.0834, **289.0707**, 265.0718, 247.0611, 127.0390, **123.0441**
M85b	EC/C–aromatic aldehyde–EC/C		12.03	C_37_H_36_O_16_	737.21033	737.21033	3.69	0	737.2103, **585.1625**, 567.1519, 507.1335, **449.1451**, 417.1219, 355.0833, 297.0984, 289.0708, 265.0723, 247.0605, 127.0389, **123.0440**
M86a	EC/C–hydroxymethylfurfural–EC/C–bisulfite	785	6.11	C_36_H_32_O_18_S	785.13679	785.13679	−1.81	1	**705.1815**, 703.1661, 551.1202, 533.1085, 485.0883, 417.1198, 289.0718, 247.0611, **127.0392, 123.0443**
M86b	EC/C–hydroxymethylfurfural–EC/C–bisulfite		6.4	C_36_H_32_O_18_S	785.13708	785.13708	−1.44	1	785.1371, **705.1753**, 703.1687, **533.1054**, 423.0729, 351.0514, 289.0717, 127.0396, **123.0446**
M86c	EC/C–hydroxymethylfurfural–EC/C–bisulfite		5.94	C_36_H_32_O_18_S	785.1371	785.1371	−1.42	1	**705.1819**, 551.1201, **417.1197**, 355.0831, 289.0716, 271.0609, 127.0396, **123.0390**
M86d	EC/C–hydroxymethylfurfural–EC/C–bisulfite		5.22	C_36_H_32_O_18_S	785.13825	785.13825	0.05	1	785.1382, **705.1797, 551.1185**, 351.0506, 289.0734, **139.0391**, 123.0444
M86e	EC/C–hydroxymethylfurfural–EC/C–bisulfite		5.09	C_36_H_32_O_18_S	785.13844	785.13844	0.29	1	**705.1846**, 583.1455, 355.0853, **127.0396, 123.0445**
M86f	EC/C–hydroxymethylfurfural–EC/C–bisulfite		10	C_36_H_32_O_18_S	785.1395	785.1395	1.64	1	785.1395, **705.1840**, 703.1654, **551.1211**, 417.1197, 271.0596, 247.0608, 127.0395, **123.0444**

a Minimum oxidation level; EC/C: epicatechin/catechin;: epicatechin/catechin; Dp: delphinidin; Cy: cyanidin; Pt: petunidin; Pn: peonidin; Mv: malvidin; glc: glucoside; Nu: nucleophile; RT: Retention time.

**Fig. 6:**
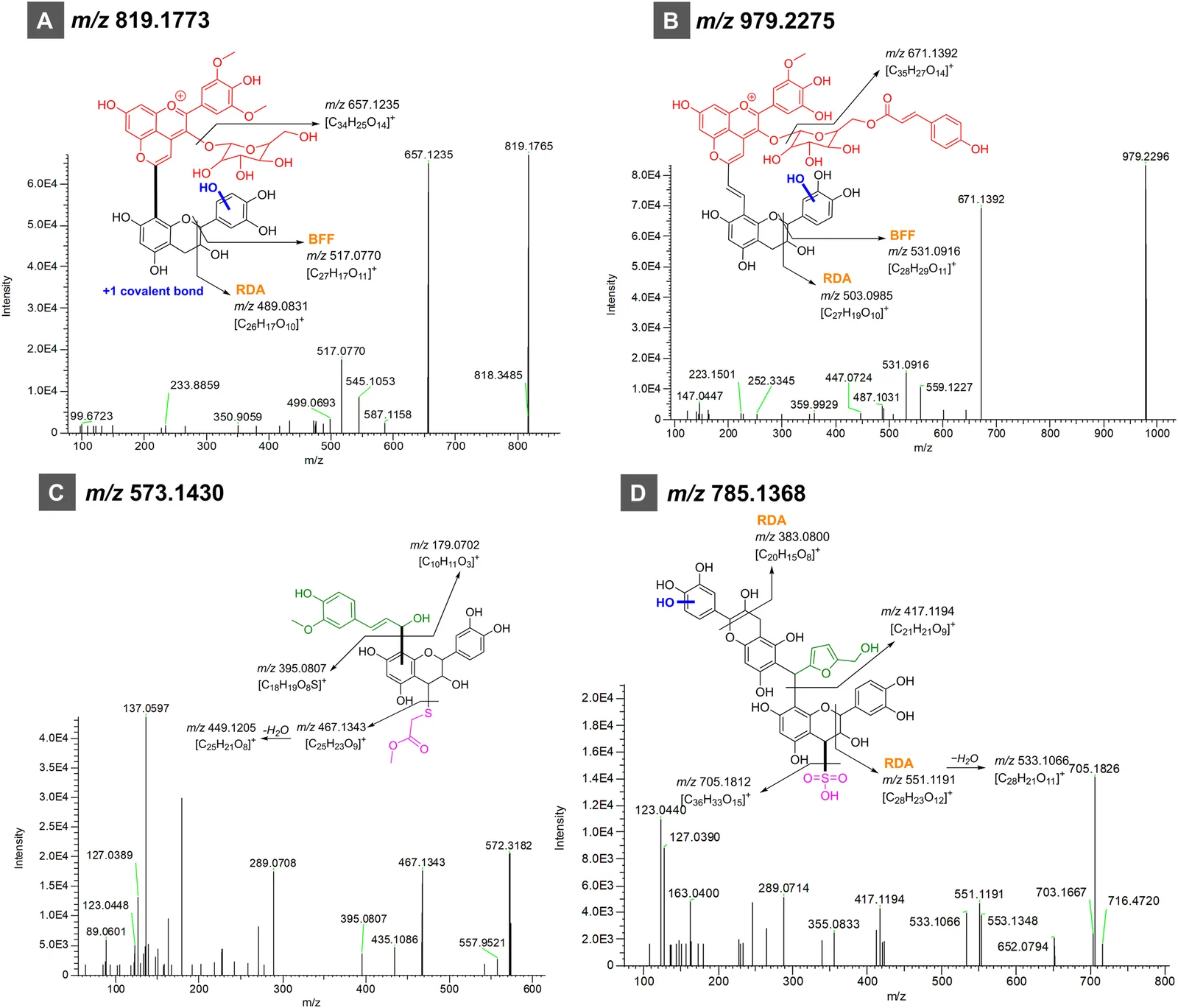
MS/MS spectrums and proposed fragmentation pathways of the oxidation markers at *m/z* 819.1773 (M66) (**A**), 979.2275 (M77) (**B**), 573.1430 (M81b) (**C**), and at *m/z* 785.1368 (M86a) (**D**).

Tannins can react with vinylflavanols, yielding a variety of pyranoanthocyanin–tannin evolution markers, highlighted as orange nodes in the MN. Among these, the known marker at *m/z* 913.2189 (M61) was assigned to EC/C–PyranoMv-3-*O*-glc-4-vinylcatechol, previously described (He et al., 2006). Its MS/MS spectra is characterized by an initial neutral loss of the glucose moiety (−162.053 Da), yielding an abundant fragment at *m/z* 751.1645, followed by RDA cleavage (−152.048 Da) leading to a fragment at *m/z* 599.1165. In addition to this known marker, 5 types of new oxidized pyranoanthocyanin–tannin markers (M62–M67) were annotated, mainly characterized by supplementary covalent bond and one or two additional OH groups, indicating oxidative modifications occurring after condensation. As an example, the oxidation marker at *m/z* 819.1773 (M66) yielded fragment ions at *m/z* 657.1235, 517.0770 and 489.0831, consistent with glucose loss (−162.058 Da) followed by a BFF (−140.048 Da) and RDA cleavages (−168.040 Da), respectively (Fig. 6A).

A second subgroup of pyranoanthocyanin–tannin markers corresponded to portisin-related structures, highlighted as blue nodes in the MN. This family comprised 13 markers in total, including previously reported (M68–M73) and new oxidized markers (M74–M78) (Table 4). Portisins are complex vinylpyranoanthocyanin-based pigments formed through reactions between pyranoanthocyanins and vinylflavanols generated from flavan-3-ols linked by an ethylene bridge (Mateus et al., 2002; Marquez et al., 2013). The known markers included vinylpyranoCy-, vinylpyranoDp-, vinylpyranoPt-, and vinylpyranoMv-3-*O*-glc–EC/C, some bearing acetylated or *p*-coumaroylated glucose moieties, confirming by neutral losses of *p*-acetylated (−204.062 Da) or *p*-coumaroylated glucose (−308.089 Da) observed in their MS/MS spectra. 6 new markers, were identified and assigned to oxidized vinylpyranoanthocyanin-derived pigments, characterized by high molecular weights, and the presence of one additional OH group. As an example, the ion at *m/z* 979.2275 (M77) exhibited a characteristic MS/MS fragmentation pattern dominated by an intense fragment ion at *m/z* 671.1392, corresponding to the neutral loss of *p*-coumaroylated glucose (−308.090 Da), followed by fragments at *m/z* 531.0916 and 503.0985 arising from BFF (−140.048 Da) and RDA cleavages, respectively (−168.041 Da) (Fig. 6B).

##### 3.1.3.4 Tannin–aromatic aldehyde interaction markers

A distinct group of tannin–aromatic aldehyde derivative markers, comprising 8 marker types (M79–M86), corresponding to 24 markers, was highlighted as light blue nodes in the MN and annotated with confident annotation levels 2–3 (Table 4). The first subgroup included adducts derived from hydroxycinnamic aldehydes and was assigned to tannin–coniferaldehyde-type markers (M79–M83). The marker at *m/z* 573.1430 (M81b) was annotated as a condensation product between an extension unit and coniferaldehyde, whereas the ions detected at *m/z* 589.137 and 605.131 likely correspond to analogous markers bearing one or two additional hydroxyl groups, formed through further oxidation of the aromatic aldehyde or the tannin moiety. Condensation with terminal units was also observed, as indicated by ions at *m/z* 469.1495 (M79) and 485.1447 (M80a). The reaction likely involves electrophilic condensation of coniferaldehyde at the C_6_ or C_8_ nucleophilic position of the flavan-3-ol unit, followed by partial reduction of the conjugated double bond or carbonyl group and rearrangement. As an example, the MS/MS spectrum of the ion at *m/z* 573.1430 (M81b) was dominated by an intense fragment ion at *m/z* 467.1343, corresponding to the neutral loss of a Nu group (−106.008 Da). Subsequent fragmentation yielded fragment ions at *m/z* 395.0807 and 289.0708, corresponding to the extension unit and flavan-3-ol unit, respectively, together with an ion at *m/z* 179.0702, which can be attributed to a coniferaldehyde-derived fragment (Fig. 6C). These fragmentation features support the involvement of aromatic aldehydes in tannin condensation reactions, which are known to occur during barrel aging (Tanaka et al., 2008).

A second subgroup of new oxidation markers were assigned to condensation products formed between tannins and hydroxymethylfurfural-derived aldehydes (M84–M86) (Table 4). The MS/MS spectrum of the marker at *m/z* 785.1368 (M86a), for example, exhibited an intense fragment ion at *m/z* 705.1812, consistent with the neutral loss of sulfur dioxide (−80.064 Da). Under wine pH conditions, bisulfite anion (HSO_3_^−^), the predominant form of SO_2_, acts as a nucleophile toward the C4 carbocation of flavan-3-ols, leading to the formation of sulfonated adducts, displaying high which display high polarity (Arapitsas et al., 2018). The subsequent loss of SO_3_ observed in the MS/MS spectra therefore supports this reaction pathway (Ma et al., 2018). Additional diagnostic fragment ions characteristic of RDA cleavage (−152.047 Da) were detected at *m/z* 551.1191, followed by loss of water yielding a fragment at *m/z* 533.1066. A further fragment ion at *m/z* 417.1194, corresponding to the loss of a flavanol unit (−288.060 Da), supports that this marker arises from structures comprising one terminal and one extension unit linked by a hydroxymethylfurfuryl bridge with an additional hydroxyl group (Fig. 6D), representing an oxidized analogue of previously described (Es-Safi et al., 2000; Nonier et al., 2010).

An overview of the annotation confidence associated with the 139 evolution markers is provided in Table 5, which summarizes their distribution across the different molecular families according to the Schymanski confidence levels.

**Table 5:** Distribution of the 139 annotated evolution markers according to Schymanski confidence level and molecular family.

Molecular family	Type of marker	Level 2–3	Level 3–4	All markers	New markers
Tannin–tannin known oxidation markers	M1–M12	23	0	23	0
Tannin–tannin new oxidation markers	M13–M34	13	17	30	30
Tannin–anthocyanin markers	M35–M60	41	0	41	5
Tannin–pyranoanthocyanin markers	M61–M78	21	0	21	13
Tannin–aromatic aldehyde markers	M79–M86	24	0	24	24

**Total**		**122**	**17**	**139**	**72**

### 3.2 Comparison of tannin evolution profiles according to cork type

**Fig. 7:**
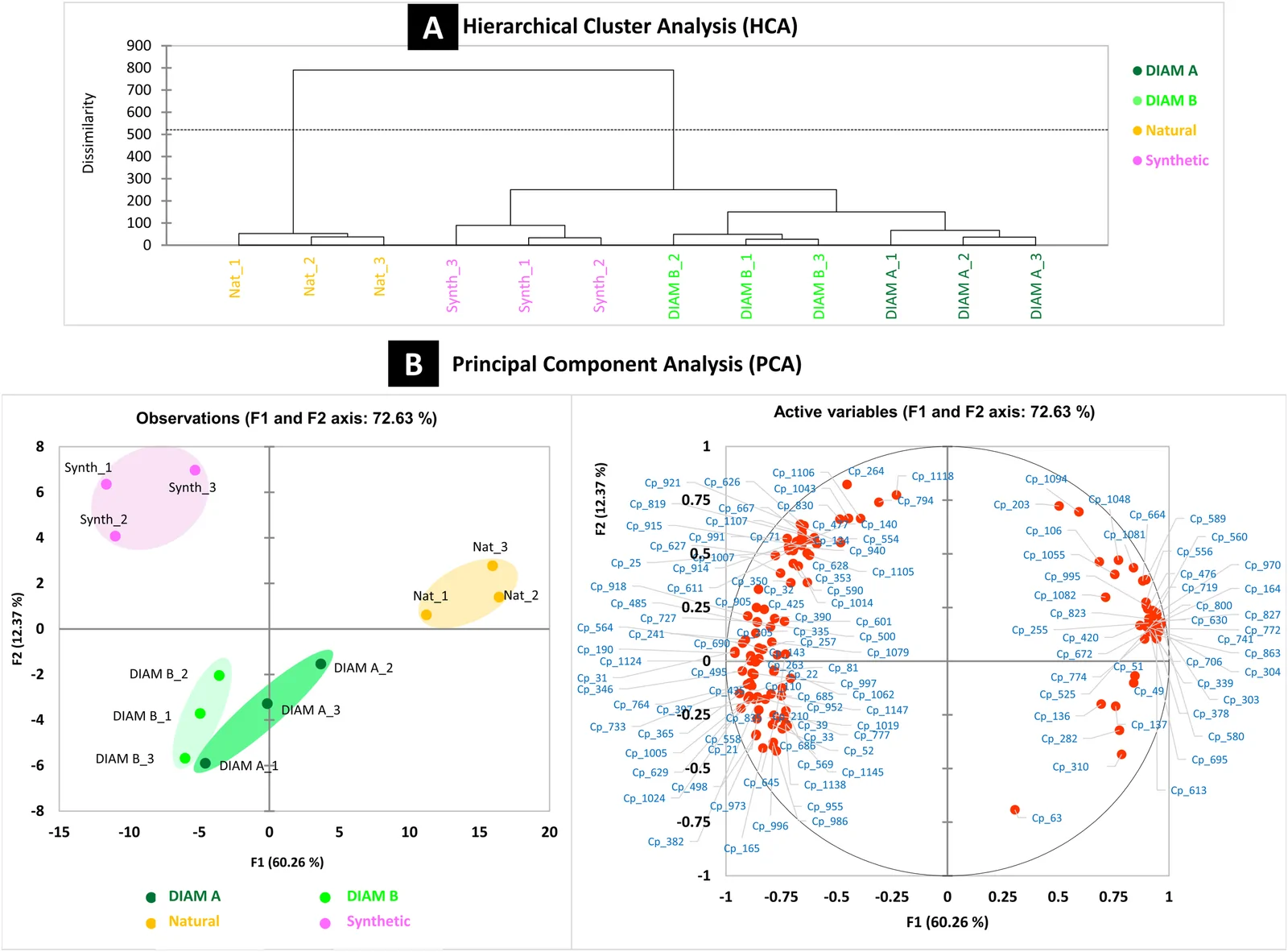
Multivariate analysis of closure-dependent discrimination based on tannin-derived molecular markers in aged wines: HCA dendrogram (A) and PCA score plot (B).

Following the structural annotation of tannin-derived markers, an exploratory statistical analysis was performed on the full-scan AcquireX™ dataset to investigate whether structurally annotated markers exhibited abundance patterns associated with closure type within this specific sample set. One-way ANOVA identified 212 variables with *p* ≤ 0.05. Detailed features and results are provided in the Supplementary Materials (Table S2). For visualization purposes, a more stringent threshold (*p* ≤ 0.03) retained 144 variables, which were further explored by PCA and HCA (Fig. 7). The results discussed below are derived from the complete set of 212 significant variables.

The HCA dendrogram (Fig. 7A) revealed a separation of the four closure groups. Natural cork samples formed a distinct cluster, separated from the other closure systems. Synthetic closures also clustered independently, whereas DIAM A and DIAM B samples grouped more closely. These patterns are consistent with the earlier metabolomic observations on the same wines (Razafindrabenja et al., 2026), suggesting that oxygen ingress could modulate the kinetics of shared chemical transformations during bottle aging.

The PCA score plot confirmed this trend (Fig. 7B). The first two principal components explained 72.63% of the total variance (F1 = 60.26%, F2 = 12.37%). Separation occurred mainly along F1, with natural cork wines on the positive side and synthetic closure wines on the negative side, suggesting distinct aging evolution between these two closure types. DIAM A and DIAM B samples clustered in the lower part of the plot (negative F2 values), high similarity but partial separation. Replicates clustered closely within each group, indicating good analytical consistency throughout the analysis.

To interpret this separation, variables selected (p ≤ 0.05) were related to previously identified marker structures based on MN and MS/MS data. As not all discriminant features could be structurally assigned and not all identified markers were discriminant, results are discussed as overall compositional trends associated with closure type.

Overall, for these studied wines, these patterns appear to be consistent with oxygen permeability effects. Each closure type was associated with a distinct profile of discriminant markers based on their relative abundances, highlighting compositional differences among closure conditions. Synthetic closures (pink), with higher oxygen permeability (OTR_T0_ = 4.6 mg/year), were associated with an increased contribution of more oxidized tannin-derived markers, which may reflect a greater extent of oxidative evolution under these closure conditions. DIAM B closure (light green), which may exhibit reduced mechanical performance after its five-year lifespan, leading to moderate permeability (OTR_T17_ = 0.66 mg/y), showed an intermediate chemical profile.

In contrast, DIAM A closure (dark green) (OTR_T17_ = 0.22 mg/y) was preferentially associated with less evolved tannin structures. This observation suggests a possible preservation effect associated with lower oxygen transfer conditions. Natural cork (orange) exhibited a more heterogeneous marker distribution, which is compatible with the greater variability generally observed for natural cork permeability.

Considering tannin–tannin oxidation markers family, several variables reached their highest relative abundance in wines sealed with highly permeable synthetic closures, while remaining comparatively minor under less permeable DIAM A closure. These include oxidation markers at *m/z* 575.1178 (M3), 591.1128 (M15), 1221.1680 (M12), 1239.1436 (M19), and 1269.1557 (M34), exhibiting oxidation levels ranging from 3 to at least 5, suggesting a higher degree of tannin oxidation for these compounds under the tested closure conditions. Conversely, less oxidized markers such as *m/z* 577.1340 (M4a), 579.1491 (M5b), and 697.1215 (M9), with one and two oxidation levels, were abundant under DIAM A closure, suggesting a protective effect of low-permeability closures against advanced tannin oxidation, which may indicate a lower progression of tannin oxidation in wines protected by lower-permeability closures. A similar trend was observed for tannin–aromatic aldehyde markers. Coniferaldehyde–EC/C-based markers at *m/z* 573.1281 (M81a), 589.1375 (M82), and *m/z* 605.1314 (M83), were relatively more abundant in wines sealed with synthetic and DIAM B closures with higher permeability.

Interestingly, tannin–anthocyanin condensation products (*m/z* 751.1881 (M36), 753.1658 (M37), 767.1813 (M38a), and 781.1986 (M40b)), did not show a direct dependence on closure oxygen permeability. This observation suggests that their formation may be mainly governed by intrinsic wine aging reactions rather than solely by oxygen exposure. Their formation is mainly driven by intrinsic condensation reactions occurring during bottle aging rather than by differences in oxygen exposure. However, more structurally complex markers, including those bearing additional IFL and additional OH groups formed by oxidation, tended to be relatively more abundant in wines sealed with highly permeable synthetic closures, indicating a profile compatible with more advanced chemical evolution under these conditions. These included markers at *m/z* 753.2023 (M47), 755.1808 (M48), 927.2331 (M57), and 1033.2439 (M59).

Most tannin–pyranoanthocyanin markers (*m/z* 833.1923 (M76a), 845.1902 (M70), and 847.2067 (M71)) were more abundant in bottles closed with synthetic closures. Their pyranoanthocyanin derivatives (P1–P10) were also detected across both synthetic and natural cork closures, suggesting a preferential formation of stable pigment structures. Although the formation of these pigments is not strictly oxidative, higher closure permeability may indirectly promote their accumulation by modulating the availability of reactive precursors, such as acetaldehyde and vinylphenols, which are progressively generated during long-term bottle aging. Overall, these observations highlight closure-related compositional trends for the wine investigated here; however, they should be considered specific to this wine matrix and aging conditions. And should not be directly extrapolated to other wines or grape varieties without further investigatio their relevance to other wine styles, grape varieties, or aging conditions remains to be established.

## 4 Conclusion

This study demonstrates the successful transfer of analytical approach combining a tannin isolation, chemical depolymerization, UHPLC–UHRMS analysis using AcquireX™ data-dependent acquisition, and Feature-Based Molecular Networking, previously validated on grape-seed extracts, to the structural characterization of condensed-tannin evolution markers in authentic highly complex 17-year-old red wines. We not only characterized both previously reported and newly markers comprehensively but also revealed their interrelationships and advanced the structural characterization of evolved tannins.

Our results also demonstrate that long-term bottle aging leads to extensive CT changes through condensation, oxidation, polymerization, and structural rearrangements. Beyond well-known oxidative and non-oxidative evolution markers such as pyranoanthocyanin pigments and A-type interflavan linkages, accumulation of higher-mass, highly oxygenated CT derivatives that have remained largely unexplored in past studies due to analytical limitations were observed. Importantly, AcquireX™ workflow proved particularly effective in detecting these late-stage oxidation products. t-SNE analyses organized thousands of features into structurally related families, facilitating marker annotation.

As a complementary result for this wine, within several marker families, wines sealed with highly permeable synthetic closures tended to exhibit higher relative abundances of markers associated with more advanced oxidation and pyranoanthocyanin–tannin structures, whereas less permeable closures were associated with a higher relative abundance of less evolved structures. These observations suggest that closure permeability may contribute to differences in tannin evolution, although the extent of this effect remains specific to the wine matrix and aging conditions investigated in this study.

Overall, while previous studies on model solutions or purified grape seed extracts have provided valuable insights, this work on highly complex wines offers a more detailed understanding of CT evolution in authentic wine matrices. Interactions between tannins, other wine constituents, and oxygen generate complex molecular architectures that only become accessible through high-resolution, untargeted, and fragmentation-informed analytical strategies. These findings provide a new platform for investigating polyphenol oxidation in complex food systems and significantly advance our understanding of wine aging chemistry.

## CRediT authorship contribution statement

Lantomalala Elsa Razafindrabenja: Investigation, Methodology, Formal analysis, Software, Data curation, Writing – original draft, Writing – review and editing, Visualization. Emmanuelle Meudec: Methodology, Formal analysis, Software, Writing – review and editing. Arnaud Verbaere: Methodology, Formal analysis, Software. Soline Caillé;: Methodology, Formal analysis. Nicolas Galy: Resources, Funding acquisition. Dimitri Tixador: Resources, Funding acquisition. Christophe Loisel: Resources, Funding acquisition. Nicolas Sommerer: Conceptualization, Methodology, Software, Supervision, Funding acquisition, Writing – review and editing, Visualization, Validation. Laetitia Mouls: Project administration, Conceptualization, Methodology, Formal analysis (strategic guidance and interpretation), Supervision, Funding acquisition, Writing – review and editing, Visualization, Validation.

## Data availability

Data will be made available on request.

## Funding sources

This work was supported by the DIAM Bouchage, under the BOTOX project.

## Declaration of competing interests

The authors declare that they have no known competing financial interests or personal relationships that could have appeared to influence the work reported in this paper.
