# Supplementary material for: Advanced structural characterization of condensed tannin evolution markers in long-aged red wines under different closure oxygen permeabilities
Source: Curr Res Food Sci. 2026 Aug 14;13:101528. doi: 10.1016/j.crfs.2026.101528 (PMC13544003; doi:10.1016/j.crfs.2026.101528)
Supplement: Supplementary file 1 [file mmc1.docx]

*Supplementary Material*

Advanced structural characterization of condensed tannin evolution markers in long-aged red wines under different closure oxygen permeabilities

Lantomalala Elsa Razafindrabenja ^a,b^, Emmanuelle Meudec ^a,b^, Arnaud Verbaere ^a,b^, Soline Caillé ^a^, Nicolas Galy ^c^, Dimitri Tixador ^c^, Christophe Loisel ^c^, Nicolas Sommerer ^a,b^ and Laetitia Mouls ^a,^ *

^a^ SPO, Univ Montpellier, INRAE, Institut Agro, Montpellier, France

^b^ INRAE, PROBE Research Infrastructure, PFP Polyphenol Analysis Facility, Montpellier, France

^c^ Diam Bouchage, Céret, France

***** Correspondence: laetitia.mouls@institut-agro.fr

Table S1. Characteristics of closures with theorical oxygen transfer rate provided by manufacturers and determined by coulometry after 17 years of storage.

| **Stopper type** | **Code** | **OTR _T0_ (mg/y)^a^** | **OTR_T17_ (mg/y)^b^** | **Durability (years)** |
| --- | --- | --- | --- | --- |
| Synthetic cork | Synth. | 4.6 | - | - |
| Microagglomerated corks | DIAM A | 0.3 | 0.22 | >30 |
|  | DIAM B | 0.4 | 0.66 | 5 |
| Natural cork | Nat. | nd: <0.1 – 283 | nd: <0.1 – 283 | - |

^a^ OTR_T0_: theorical oxygen transfer rate provided by manufacturer and determined by chemiluminescence (PreSens) for synthetic and microagglomerated stoppers or coulometry for screw caps. nd: non-determined; natural cork has a high intrinsic structural variability that impacts OTR (Crouvisier-Urion *et al.*, 2018). ^b^ OTR_T17_: oxygen transfer rate measured after 17 years of aging.

Retention Time (min)

| Week | Gallic acid | Cafeic acid | Epicatechin | Rutin | Quercetin |
| --- | --- | --- | --- | --- | --- |
| 3 | 1 | 5.94 | 7.55 | 12.06 |  |
| 10 | 0.87 | 5.57 | 7.24 | 11.69 |  |
| 15 | 0.88 | 5.61 | 7.3 | 11.84 |  |
| 18 | 0.89 | 5.66 | 7.31 | 11.91 |  |
| 22 | 0.88 | 5.68 | 7.37 | 11.92 |  |
| 25 | 0.88 | 5.69 | 7.44 | 12.01 |  |
| 29 | 0.89 | 5.72 | 7.46 | 12.05 |  |
| 38 | 0.88 | 5.68 | 7.42 | 12 |  |
| 45 | 0.89 | 5.69 | 7.39 | 12.03 |  |
| 66 | 0.89 | 5.72 | 7.47 | 12.1 |  |
| 67 | 0.89 | 5.7 | 7.48 | 12.12 |  |
| 70 | 0.88 | 5.71 | 7.47 | 12.11 |  |
| 73 | 0.87 | 5.91 | 7.35 | 11.42 |  |
| 74 | 0.97 | 5.91 | 7.4 | 11.47 |  |
| 81 | 0.89 | 5.76 | 7.49 | 12.11 |  |
| 84 | 0.92 | 5.81 | 7.59 | 12.18 |  |
| 92 | 0.9 | 5.77 | 7.48 | 12.13 |  |
| 95 | 0.91 | 5.77 | 7.48 | 12.15 |  |
| 100 | 0.89 | 5.77 | 7.5 |  | 15.32 |
| 119 | 0.9 | 5.76 | 7.54 |  | 15.38 |
| 134 | 0.926717 | 5.86005 | 7.60005 |  |  |
| 143 | 0.9 | 5.79 | 7.57 |  | 15.37 |
| 147 | 1.23 | 6.46 | 8.26667 |  | 15.5667 |
| 148 | 1.03005 | 6.19672 | 8.04338 |  | 15.4834 |
| 169 | 0.9 | 5.78 | 7.56 |  | 15.37 |
| 196 | 0.88 | 5.64 | 7.35 |  | 15.31 |
| 199 | 0.87335 | 5.61002 | 7.32335 |  | 15.24 |
| 205 | 0.87 | 5.6 | 7.28 |  | 15.23 |
| 210 | 0.866733 | 5.57673 | 7.33007 |  | 15.2534 |
| 214 | 0.86 | 5.6 | 7.34 |  | 15.25 |
| 231 | 0.86 | 5.63 | 7.33 |  | 15.22 |
| 238 | 0.89 | 4.97 | 6.46 |  | 14.44 |
| 239 | 0.99 | 4.98 | 6.46 | 11.16 |  |
| 240 | 0.900017 | 5.01335 | 6.47335 | 11.14 |  |
| 243 | 0.89 | 4.95 | 6.41 | 11.05 |  |
| 244 | 0.89 | 4.94 | 6.39 | 11.06 |  |
| 245 | 0.89 | 4.94 | 6.4 | 11.05 |  |
| 246 | 0.890017 | 4.94668 | 6.40002 | 11.0567 |  |
| 252 | 0.8934 | 4.96007 | 6.41673 | 11.0734 |  |
| 255 | 0.89005 | 4.95338 | 6.39338 | 11.0234 |  |
| 260 | 0.89335 | 4.96668 | 6.41668 | 11.0534 |  |
| 266 | 0.886717 | 4.94005 | 6.38005 | 11.0401 |  |
| 274 | 0.9 | 4.97 | 6.42 | 11.08 |  |
| 281 | 0.9 | 4.96 | 6.41 | 11.08 |  |
| 288 | 0.9 | 4.97667 | 6.42 | 11.0967 |  |
| 289 | 0.9 | 4.95667 | 6.4 | 11.1 |  |
| 308 | 0.87 | 4.92 | 6.37 | 11.06 |  |
| 316 | 0.9 | 4.98 | 6.46 | 11.16 |  |
| 343 | 0.91 | 5.02 | 6.51 | 11.26 |  |
| 345 | 0.903333 | 5 | 6.48 | 11.2233 |  |
| 350 | 0.93335 | 5.04335 | 6.52002 | 11.29 |  |

Signal/Noise (UV 280 nm)

| Week | Gallic acid | Cafeic acid | Epicatechin | Rutin | Quercetin |
| --- | --- | --- | --- | --- | --- |
| 3 | 4590 | 3980 | 1050 | 1730 |  |
| 10 | 4800 | 4340 | 1360 | 2060 |  |
| 15 | 4260 | 3890 | 1170 | 1820 |  |
| 18 | 4970 | 4460 | 1300 | 2080 |  |
| 22 | 4890 | 4220 | 1240 | 43.7 |  |
| 25 | 4640 | 3930 | 1170 | 1880 |  |
| 29 | 4460 | 3910 | 1100 | 1800 |  |
| 38 | 4700 | 3950 | 1180 | 1860 |  |
| 45 | 4330 | 3780 | 1060 | 1720 |  |
| 66 | 4630 | 3930 | 1130 | 1800 |  |
| 67 | 4720 | 3910 | 1120 | 1820 |  |
| 70 | 4710 | 3970 | 1130 | 1730 |  |
| 73 | 4610 | 4400 | 1450 | 2160 |  |
| 74 | 4640 | 4410 | 1440 | 2150 |  |
| 81 | 4920 | 4210 | 1140 | 1610 |  |
| 84 | 4760 | 4050 | 1110 | 1760 |  |
| 92 | 4840 | 3970 | 1080 | 1710 |  |
| 95 | 4810 | 3910 | 1060 | 1730 |  |
| 100 | 5380 | 2820 | 1210 |  | 13.6 |
| 119 | 4930 | 2570 | 1070 |  | 525 |
| 134 | 4770.43 | 2496.75 | 1135.07 |  |  |
| 143 | 5250 | 2760 | 1150 |  | 660 |
| 147 | 4523.58 | 3420.72 | 1125.34 |  | 594.221 |
| 148 | 5215.92 | 3137.59 | 1072.65 |  | 526.009 |
| 169 | 5300 | 2680 | 1160 |  | 615 |
| 196 | 5440 | 2630 | 1150 |  | 741 |
| 199 | 5562.53 | 2736.34 | 1214.67 |  | 445.854 |
| 205 | 5540 | 2700 | 1210 |  | 1200 |
| 210 | 5231.07 | 2466.21 | 1153.17 |  | 1682.34 |
| 214 | 5450 | 2530 | 1200 |  | 1710 |
| 231 | 5274.06 | 2534.82 | 1172.31 |  | 867.261 |
| 238 | 4280 | 2390 | 982 |  | 516 |
| 239 | 6070 | 3050 | 1790 | 1620 |  |
| 240 | 3831.34 | 3604.46 | 1099.08 | 2088.09 |  |
| 243 | 4090 | 3710 | 1190 | 2100 |  |
| 244 | 4060 | 3760 | 1170 | 2070 |  |
| 245 | 4150 | 3830 | 1190 | 2070 |  |
| 246 | 4035.85 | 3704.72 | 1152.67 | 2011.77 |  |
| 252 | 4104.81 | 3748.52 | 1163.37 | 2110.13 |  |
| 255 | 3896.4 | 3549.1 | 1120.89 | 2070.83 |  |
| 260 | 3831.88 | 3494.69 | 1079.35 | 1940.73 |  |
| 266 | 3966.17 | 3588.54 | 1133.74 | 1966.88 |  |
| 274 | 4200 | 3750 | 1160 | 1990 |  |
| 281 | 4120 | 3820 | 1170 | 2440 |  |
| 288 | 3585.34 | 3319.65 | 992.484 | 3207.48 |  |
| 289 | 3797.63 | 3431.15 | 1064.2 | 1946.59 |  |
| 308 | 82.1 | 72.9 | 22.7 | 42.7 |  |
| 316 | 4000 | 3550 | 1040 | 1780 |  |
| 343 | 5720 | 4970 | 1410 | 2540 |  |
| 345 | 5408.53 | 4700.3 | 1389.07 | 2389.52 |  |
| 350 | 4447.37 | 5130.21 | 1554.6 | 2781.25 |  |

UV area (280 nm)

| Week | Gallic acid | Cafeic acid | Epicatechin | Rutin | Quercetin |
| --- | --- | --- | --- | --- | --- |
| 3 | 147600 | 184000 | 55970 | 84620 |  |
| 10 | 144500 | 184800 | 57020 | 86770 |  |
| 15 | 142000 | 181300 | 56080 | 85810 |  |
| 18 | 144400 | 183900 | 56840 | 88190 |  |
| 22 | 145000 | 183900 | 56790 | 88520 |  |
| 25 | 143100 | 179100 | 56210 | 86000 |  |
| 29 | 144000 | 181300 | 55790 | 85060 |  |
| 38 | 141600 | 177800 | 55480 | 83980 |  |
| 45 | 142000 | 179500 | 55160 | 84840 |  |
| 66 | 147400 | 186200 | 56940 | 86060 |  |
| 67 | 148400 | 186700 | 57200 | 87380 |  |
| 70 | 142600 | 181000 | 55290 | 84430 |  |
| 73 | 160600 | 211300 | 67450 | 105100 |  |
| 74 | 162700 | 211600 | 67780 | 106200 |  |
| 81 | 143100 | 180500 | 54200 | 76550 |  |
| 84 | 144700 | 181900 | 55610 | 83350 |  |
| 92 | 148400 | 184700 | 56320 | 85720 |  |
| 95 | 143800 | 178700 | 54550 | 84170 |  |
| 100 | 132700 | 106700 | 50380 |  | 33640 |
| 119 | 124400 | 101500 | 47570 |  | 20030 |
| 134 | 136288 | 110368 | 53194.6 |  |  |
| 143 | 134400 | 108300 | 51100 |  | 24340 |
| 147 | 125092 | 107028 | 48471.7 |  | 19870.1 |
| 148 | 122162 | 98467.1 | 44628.1 |  | 17346.4 |
| 169 | 126000 | 102100 | 48410 |  | 21540 |
| 196 | 137000 | 112400 | 52990 |  | 27900 |
| 199 | 138545 | 113915 | 53774 |  | 16703.7 |
| 205 | 138400 | 113900 | 53940 |  | 52050 |
| 210 | 133121 | 108951 | 52144.2 |  | 73519.5 |
| 214 | 139800 | 133300 | 54570 |  | 72880 |
| 231 | 133121 | 108951 | 52144.2 |  | 73519.5 |
| 238 | 141000 | 116600 | 54630 |  | 28140 |
| 239 | 236700 | 174000 | 117100 | 104600 |  |
| 240 | 125001 | 171403 | 59672.1 | 110649 |  |
| 243 | 125400 | 171400 | 59910 | 107000 |  |
| 244 | 124500 | 170500 | 59270 | 105900 |  |
| 245 | 127000 | 173500 | 60360 | 107100 |  |
| 246 | 125047 | 171730 | 59409.9 | 104542 |  |
| 252 | 124303 | 169571 | 59052.4 | 107492 |  |
| 255 | 124090 | 169924 | 59133.3 | 111048 |  |
| 260 | 123318 | 169009 | 57662.1 | 104766 |  |
| 266 | 124292 | 170452 | 59124 | 104761 |  |
| 274 | 121000 | 163700 | 56070 | 96670 |  |
| 281 | 123600 | 168300 | 58150 | 120200 |  |
| 288 | 122782 | 168231 | 57643.2 | 188657 |  |
| 289 | 122410 | 165211 | 57165 | 106118 |  |
| 308 | 114869 | 170357 | 60131.8 | 115059 |  |
| 316 | 125300 | 169500 | 57550 | 98260 |  |
| 343 | 125000 | 171100 | 56990 | 99680 |  |
| 345 | 122014 | 168272 | 57103.1 | 96684.1 |  |
| 350 | 122485 | 177796 | 60195.2 | 104287 |  |

MS area (EIC)

| Week | Gallic acid | Cafeic acid | Epicatechin | Rutin | Quercetin |
| --- | --- | --- | --- | --- | --- |
| 3 | 651300000 | 1313000000 | 1526000000 | 1753000000 |  |
| 10 | 472500000 | 1162000000 | 1271000000 | 1470000000 |  |
| 15 | 570000000 | 1345000000 | 1386000000 | 1751000000 |  |
| 18 | 537200000 | 1339000000 | 1512000000 | 1864000000 |  |
| 22 | 640600000 | 1350000000 | 1520000000 | 1890000000 |  |
| 25 | 467800000 | 1258000000 | 1363000000 | 1703000000 |  |
| 29 | 640400000 | 1174000000 | 1315000000 | 1549000000 |  |
| 38 | 567300000 | 1187000000 | 1446000000 | 1595000000 |  |
| 45 | 522000000 | 1202000000 | 1373000000 | 1654000000 |  |
| 66 | 505000000 | 951000000 | 983500000 | 530100000 |  |
| 67 | 652800000 | 1318000000 | 1517000000 | 1790000000 |  |
| 70 | 455000000 | 1138000000 | 1318000000 | 1415000000 |  |
| 73 | 538000000 | 1221000000 | 1368000000 | 1629000000 |  |
| 74 | 535600000 | 1249000000 | 1440000000 | 1787000000 |  |
| 81 | 534100000 | 1309000000 | 1348000000 | 1718000000 |  |
| 84 | 468800000 | 1303000000 | 1561000000 | 1936000000 |  |
| 92 | 743300000 | 1568000000 | 1855000000 | 2220000000 |  |
| 95 | 574300000 | 1308000000 | 1623000000 | 1848000000 |  |
| 100 | 512800000 | 848400000 | 1451000000 |  | 921500000 |
| 119 | 433700000 | 733770000 | 1204000000 |  | 573100000 |
| 134 | 473825294 | 840643981 | 1468283497 |  |  |
| 143 | 521800000 | 800000000 | 1415000000 |  | 725300000 |
| 147 | 1022878713 | 1625092349 | 2880842564 |  |  |
| 148 | 830972017 | 1479364187 | 2594829392 |  |  |
| 169 | 557000000 | 815300000 | 1446000000 |  | 808800000 |
| 196 | 495200000 | 791700000 | 1484000000 |  | 811500000 |
| 199 | 634166386 | 782788691 | 1327642917 |  | 454288190 |
| 205 | 494900000 | 805400000 | 1380000000 |  | 2819000000 |
| 210 | 324002745 | 683397880 | 1182132963 |  |  |
| 214 | 465100000 | 739600000 | 1322000000 |  | 2507000000 |
| 231 | 294274250 | 670666952 | 1168538548 |  |  |
| 238 | 490000000 | 728900000 | 1221000000 |  | 1247000000 |
| 239 | 837100000 | 1015000000 | 2403000000 | 1708000000 |  |
| 240 | 406769424 | 1029948665 | 1305161758 | 1679626113 |  |
| 243 | 403400000 | 1053000000 | 1312000000 | 1688000000 |  |
| 244 | 410700000 | 1062000000 | 1304000000 | 1766000000 |  |
| 245 | 428830000 | 1075000000 | 1372000000 | 1792000000 |  |
| 246 | 509407385 | 1206961914 | 1551340649 | 1994399364 |  |
| 252 | 474770238 | 953285712 | 1225549139 | 1648588424 |  |
| 255 | 381947369 | 874674998 | 1153004090 | 1540222570 |  |
| 260 | 504664790 | 1076376784 | 1391055470 | 1618603626 |  |
| 266 | 408615888 | 1022318959 | 1375230987 | 1732095186 |  |
| 274 | 563700000 | 1165000000 | 1256000000 | 2026000000 |  |
| 281 | 474000000 | 1251000000 | 1594000000 | 2327000000 |  |
| 288 | 414664085 | 1154720663 | 1491345685 | 3069524902 |  |
| 289 | 597320757 | 1147543652 | 1494815853 | 1695298231 |  |
| 308 | 315605827 | 973138735 | 1221176334 | 1829679353 |  |
| 316 | 377800000 | 1110000000 | 1467000000 | 1951000000 |  |
| 343 | 345200000 | 797200000 | 1093000000 | 1480000000 |  |
| 345 | 407472483 | 984784468 | 1438144584 | 1929828510 |  |
| 350 | 324244664 | 1015727711 | 1276663728 | 1879922225 |  |

**S2. Annual monitoring of analytical system stability using retention time, UV peak areas, signal-to-noise ratios, and MS response**

**S3. MZmine 4.7.29 software processing parameters**

*Peak Detection (0.3-21 min)*

Scan MS1 centroid; noise level: 5^E^4

Scan MS2 centroid; noise level: 1^E^2

*Chromatogram Builder (0.3-21 min)*

Scan MS1

Min group size of scans: 5

Group intensity threshold: 1.5^E^5

Min highest intensity: 1.5^E^5

Scan to scan accuracy: 0.0050 *m/z* or 10.0 ppm

*Chromatogram Deconvolution/ Local minimum feature resolver*

MS/MS scan pairing: true

Retention time tolerance: 0.2 min

MS1 to MS2 precursor tolerance: 0.0050 *m/z* or 10.0 ppm

Minimum relative feature height: 25%

Minimum required signal: 1

Dimension: Retention time

Chromatographic threshold: 90%

Minimum search range RT: 0.050 min

Minimum relative height: 0%

Minimum absolute height: 1.5^E^5

Min ratio of peak top/edge: 1.7

Peak duration range: 0.02-10.0 min

Min # of data points: 5

*^13^C isotope filter (formerly: isotope grouper)*

*m/z* tolerance: 0.0050 *m/z* or 10.0 ppm

RT tolerance: 0.05 min

Maximum charge: 2

Representative isotope: Most intense

Never remove feature with MS^2^: true

*Join aligner*

*m/z* tolerance: 0.0050 *m/z* or 10.0 ppm

Weight for *m/z*: 75

RT tolerance: 0.05 min

Weight for RT: 25

*Filtering feature list rows*

Validate ^13^C isotope pattern: true

*m/z* tolerance: 0.0050 *m/z* or 10.0 ppm

Remove redundant isotope row: true

Max charge: 2

Never remove feature with MS^2^: true

*Feature list blank subtraction*

Minimum # of detection in blanks: 3

Fold change increase: true (300%)

*Gap filling*

Intensity tolerance: 10%

*m/z* tolerance: 0.0050 *m/z* or 10.0 ppm

RT tolerance: 0.05 min

Minimum scan (data points): 1

*Duplicate feature list rows filter*

Filter mode: New average

*m/z* tolerance: 0.0050 *m/z* or 10.0 ppm

RT tolerance: 0.05 min

*Export molecular networking files*

Filter rows: Only with MS^2^

Merging m/z tolerance: 0.0050 *m/z* or 10.0 ppm

Feature intensity: peak area

CSV export: simple

**S4. Parameters use for Metgem 1.5.2**

*Cosine Score computing*

*m/z* tolerance: 0.02 Da

Minimum matched peaks: 6

Filtering: true

Keep peaks higher than: 50 Da

Keep peaks outside of the: ± 17 Da

Keep each peak in top: 6

*Network visualization*

Maximum neighbor number (TopK): 10

Minimal cosine mscore value: 0.7

Maximum connected component size: 1000

Scale: 30

Gravity: 1.0

*t-SNE visualization*

At least:1 (cosine score above 0.7)

Minimal cosine score value: 0.7

Number of iterations: 1000

Perplexity: 6

Learning rate: 200

Early exaggeration: 12

Use barnes-hut approximation: true

Angle: 0.7

**S5. Tannin concentrations (g.L^−1^) in red wine samples sealed with different types of cork.**

Tannin concentration (g.L⁻¹)

|  | Moyenne |
| --- | --- |
| Synth. | 2.72 |
| DIAM A | 2.365 |
| DIAM B | 2.25 |
| Nat. | 2.50667 |
